# Aging kidney is associated with metabolic rewiring and epigenetic reprogramming

**DOI:** 10.1007/s11357-025-02071-0

**Published:** 2026-01-07

**Authors:** Pragney Deme, Rhea Bhargava, Heddwen L. Brooks, Norman J. Haughey, Prerna Kumar

**Affiliations:** 1 Department of Physiology, Tulane University School of Medicine, 1430 Tulane Ave, New Orleans, LA 70112 USA; 2 Department of Medicine Nephrology and Hypertension, School of Medicine, Tulane University, New Orleans, LA 70112 USA

**Keywords:** Renal aging, Metabolomics, Metabolite cofactors for epigenetic enzymes, Epimetabolites, Sex differences

## Abstract

Understanding the direct connections between metabolism and chromatin dynamics may uncover potential mechanisms involved in the aging process of renal physiology. Despite known differences in incidence and aging renal disease, how biological aging intersects with renal metabolism and epigenetics in a sex-specific context remains poorly understood. Here, we determined the effect of age on renal metabolic pathways and metabolite cofactors of epigenetic modifiers in a sex-specific manner. We measured metabolites in kidney homogenates from young and aged mice by HPLC-TripleTOF (LC–MS). The major metabolic adaptations observed with aging include increased glycolysis, decreased fatty acid oxidation, mitochondrial dysfunction, oxidative stress, and impaired metabolic waste clearance in 24-month-old (aged) mice compared to 4-month-old (young) sex-matched mice. Additionally, we found elevated levels of methylation and acetylation of intermediate metabolites also known as ‘epimetabolites’ in aged mice. Furthermore, age-related alterations were detected in metabolites (acetyl-coenzyme A, flavin adenine dinucleotide, and α-ketoglutarate) that are essential cofactors for the activities of epigenetic enzymes. Sex-specific changes were observed with age such as, significantly enhanced amino acid catabolism and tryptophan metabolism and reduced lysophospholipase activity and ammonia clearance in aged female vs aged male mice. Our results reveal age- and sex-associated alterations in renal metabolic pathways, characterized by an increase in epigenetically modified intermediate metabolites with aging. These findings suggest a complex interplay between renal metabolomics and epigenetics and offer new insights into the mechanisms underlying sex-specific renal physiology of aging kidneys.

**Graphical Abstract:**

**Figure:**
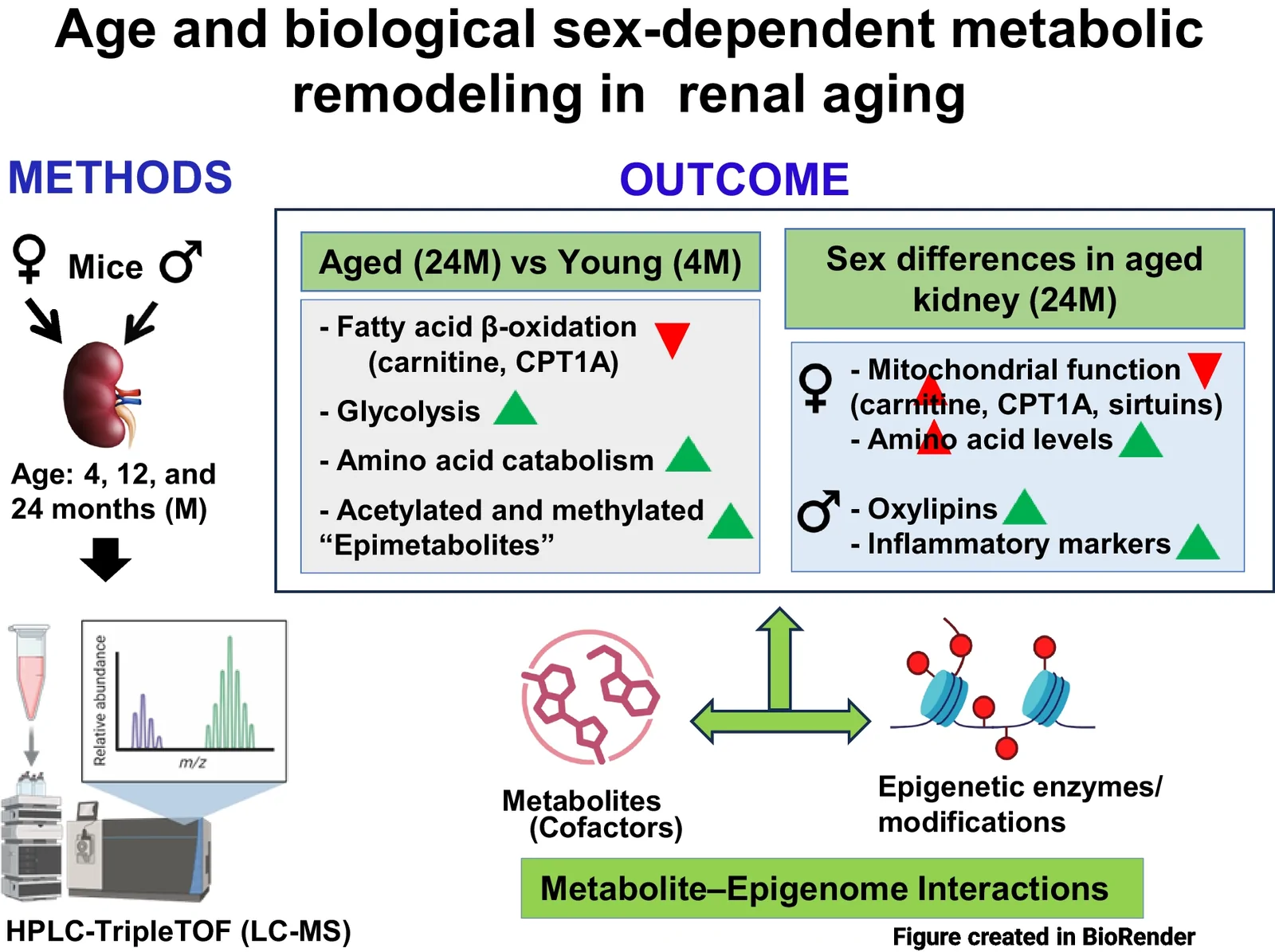

**Supplementary Information:**

The online version contains supplementary material available at https://doi.org/10.1007/s11357-025-02071-0.

## Introduction

Aging is a multifaceted biological phenomenon characterized by pronounced systemic dysregulation that notably affects various organs, including the kidneys. More than 1 in 7 adults in the United States of America, about 35.5 million people, are estimated to have chronic kidney disease (CKD) and it is more common in people aged 65 years or older (34%) than in people aged 45–64 years (12%) or 18–44 years (6%) [1]. Evidence indicates that aging kidneys present a heightened susceptibility to CKD due to an array of comorbidities such as hypertension, diabetes, obesity, and cardiovascular diseases [2, 3]. Renal aging is marked by significant structural and functional alterations, including a progressive decline in glomerular filtration rate (GFR), glomerulosclerosis, tubular dysfunction and interstitial fibrosis [4–6]. These changes contribute to diminished renal resilience and correspondingly increase the prevalence of CKD as age advances [7]. The phenomenon of renal aging can be conceptualized as a complex interplay among genetic, epigenetic, and environmental factors, all of which are intricately connected to systemic pathophysiological changes. In parallel, metabolic reprogramming has emerged as a critical axis influencing organ function during aging. Particularly, the kidneys serve as a vital organ for studying age-associated metabolic changes, given their role in modulating systemic metabolite levels through various physiological processes such as tubular secretion, glomerular filtration, and anabolism versus catabolism [8].

Furthermore, a growing body of clinical and preclinical evidence has elucidated the significant impact of biological sex on renal pathophysiology, disease outcomes, and therapeutic responses. Several meta-analyses and preclinical models of renal injury have highlighted that adult males exhibit more pronounced alterations in renal structure and function, which consequently precipitates a more severe trajectory of CKD progression when compared to age-matched females [9–11]. Notably, in women, kidney disease progression tends to worsen after menopause [12]. Sex-linked chromosomal mechanisms and sex-specific hormonal fluctuations are increasingly recognized for their roles in metabolic processes, which have been correlated with the emergence of age-related disorders such as metabolic syndrome, obesity, and diabetes [13]. Nonetheless, existing literature provides limited insight into sex differences regarding renal metabolite processing, gene expression profiles, disease susceptibility, and pharmacological responses.

Recent studies have implicated epigenetic modifications, including DNA methylation [14, 15] and histone modifications [16] in both the initiation and progression of age-related renal changes. Importantly, aberrant epigenetic modifications are increasingly being tied to age-related kidney disorders. Components of epigenetic programming often necessitate intermediates derived from cellular metabolic pathways to facilitate enzymatic functions. Recent research efforts have elucidated connections between metabolism and chromatin dynamics, providing potential explanations for many instances of metabolic dysfunction observed in aging. This chromatin regulation is characterized by epigenetic enzymes that utilize metabolite cofactors to enact modifications on DNA or histones; for instance, acetyltransferases require acetyl-coenzyme A (ac-CoA), methyltransferases rely on S-adenosyl methionine (SAM), deacetylases utilize nicotinamide adenine dinucleotide (NAD), and demethylases depend on flavin adenine dinucleotide (FAD) or α-ketoglutarate (αKG) as essential cofactors [17].

In conjunction with the metabolic control exerted over the epigenome, metabolic pathways themselves are subject to epigenetic regulation as a response to environmental stimuli. Our recent investigations have revealed biological sex- and age-associated variations in the regulation of renal injury markers and epigenetic enzyme activity in murine models [18, 19]. However, the literature remains sparse regarding the age- and sex-related differential regulation of renal metabolic pathways and their associated metabolites that serve as cofactors for diverse epigenetic enzymes in the renal epigenome. Thus, the present study seeks to employ metabolomic analyses to comprehensively examine alterations in kidney metabolites, metabolic pathways, and the role of metabolite cofactors in the epigenomic regulation during the aging process.

## Methods

### Sex as a biological variable

In this study both male and female mice kidney tissues were used for experimental analysis and tissues were age- and sex-matched. Biological sex has been considered in the analysis of the human urine samples, and their interpretation.

### Mice tissue

Kidneys of C57BL/6JN male and female mice of 4M (young), 12M (middle aged), and 24M (aged) were kindly provided by the National Institute of Aging (NIA) Aged Rodent Tissue Bank (https://www.nia.nih.gov/research/dab/aged-rodent-tissue-bank).” The animals are euthanized by CO_2_ asphyxiation then unfixed tissue and organs are harvested, flash frozen in liquid nitrogen, and stored at –80 °C.

### Extraction and profiling of metabolites by HPLC-TripleTOF-MS/MS

For untargeted metabolomics, we used n = 10 mice per group for the 4M and 24M male and female cohorts and 12M male and female groups consisted of n = 8 mice. Kidney tissues were homogenized in double distilled water (ddH_2_O). Metabolites from tissue homogenates (protein normalized ~ 250 ug/ml) were extracted by protein precipitation as described earlier [20]. Briefly, tissue homogenates were protein precipitated using 1 mL of 70% ice cold methanol (0.5% 1 N HCl), containing pre-spiked 12 isotopically (^2^H, ^15^C) labeled internal standards (IS) as described previously [20]. Chromatographic separation of extracted metabolites was achieved on Pentafluorophenyl (PFP) HPLC column, 150X2 mm, 3 µM particle size, (Phenomenex, CA, USA). Triple time of flight (Q-TOF, 5600 series) mass spectrometer (AB Sciex, ON, Canada) was used to detect metabolites in an Information Dependent Acquisition (IDA-HRMS/MS) approach, which attains accurate m/z (mass to charge ratio) of both the pre-cursor and fragments of each metabolite in the samples within a given mass range of 30–900 m/z. The acquired data were processed using Sciex OS -Q1.5 data analysis software integrated with the NIST (2017) tandem mass spectral library, and the Sciex accurate MS/MS spectral library 2.0 for peak detection, peak alignment, spectral processing, feature identification, and peak quantification. A targeted list of metabolites was prepared using pooled kidney sample extracts and validated through sequential injections with stringent criteria, as described earlier [20]. This targeted list was then used to search for metabolites in individual tissue sample extracts for quantitative assessment.

### Kidney total protein, nuclear, and histone extract preparation and immunoblot assay

Frozen kidney tissues were homogenized in RIPA (Radio-Immunoprecipitation Assay) lysis buffer (Santa Cruz Biotechnology, Santa Cruz, CA) and the homogenate was centrifuged at ∼14,000 × g for 15 min. The supernatant was stored at −80^0^C. Nuclear and histone extracts were prepared from mice kidney tissues using EpiQuik Nuclear Extraction Kit and EpiQuik and Total Histone Extraction Kit, respectively, from EpigenTek (Farmingdale, NY) following manufacturers protocol. The protein concentration of the extract was measured with Bradford protein detection kit (Bio-Rad, Hercules, CA). The protein extract (50–60 µg) was separated using 10% sodium dodecyl sulfate–polyacrylamide gel electrophoresis (SDS-PAGE) as previously described [18, 21]. Visualization of protein bands and densitometry analysis was performed essentially as described earlier [19]. The catalog number and the dilutions of the antibodies used in Western blot are listed as follows: cPLA2 (2832; 1:1000), LDHA (2012; 1:1000), CPT1 (12252; 1:1000), HDAC1 (34589S; 1:1000), HDAC2 (57156S; 1:1000), HDAC3 (3949S; 1:1000), HRP-conjugated anti-rabbit IgG (7074; 1:5000) from (Cell Signaling Technology, Danvers, MA); β-actin (sc-47778-HRP; 1:5000), and HRP-conjugated anti-mouse IgG (sc-516102; 1:5000) from Santa Cruz Biotechnology, Santa Cruz, CA).

### Total HAT, HDAC, and SIRT activity assay

Total HAT and HDAC activities were measured in the renal nuclear extracts (20 μg) using colorimetric EpiQuik HAT and Epigenase HDAC activity/inhibition assay kits, respectively, from Epigentek (Farmingdale, NY) as described in our previous studies [21, 22]. SIRT activity was measured by Epigenase™ Universal SIRT Activity/Inhibition Assay Kit from Epigentek following manufacturers protocol.

### Quantification of global H3 lysine acetylation

Following Epigentek’s protocol, renal total H3 acetylation (ac) levels were quantified in histone extracts using EpiQuik™ Total Histone H3 Acetylation Detection Fast Kit (Colorimetric). In the assay, the acetyl histone H3 is captured to the strip wells coated with an anti-acetyl histone H3 antibody and detected with a labeled detection antibody. The ratio of acetyl histone H3 is proportional to the intensity of absorbance read at 450 nm and the acetyl histone H3 levels were calculated using a standard curve.

### Total DNMT and DNA demethylase (TET) activity assay

Total DNMT activity and TET enzymatic activity was measured in the renal nuclear extract by ELISA kits EpiQuik™ DNMT Activity and Epigenase™5mC Hydroxylase TET Activity kits (Colorimetric), respectively, purchased from Epigentek following manufacturers protocol.

### Total histone demethylases (HDMs) activity assay

Enzymatic activities of lysine specific demethylases, JMJD3 and LSD1 were measured by Epigenase™ JMJD3/UTX Demethylase Activity and LSD1 Demethylase Activity Assay Kits, respectively, from Epigentek essentially following the manufacturers protocol.

### Urine collection and processing

Spot urine samples were collected from human subjects during routine clinic visits. Subjects who provided informed consent were asked to donate urine samples for laboratory research at intervals of no less than eight weeks. Samples consisted of approximately 3 oz of urine, collected in sterile urine cups and immediately transported to the laboratory for processing as previously described in our earlier studies [23]. Briefly, fresh urine samples (~ 50 ml) were centrifuged for 10 min at 1,500 revolutions per minute. The supernatant was separated and aliquoted and stored at −80C. The supernatant was used for measurement of metabolites in this study. Exclusion criteria for the sample collection: Terminally ill patients and vulnerable subjects (e.g., pregnant women, prisoners, or individuals with cognitive impairment). Data collection was approved by the institutional review board at Tulane University School of Medicine. Informed assent and consent were obtained from the patients and caregivers before enrolling in the study, in accordance with principles of the Declaration of Helsinki.

### Quantification of urine metabolites

Urinary metabolites were measured utilizing commercial kits as described below and the results were normalized with creatinine concentration.

#### Creatinine

Creatinine concentration was measured in the urine samples utilizing QuantiChromTM Creatinine Assay Kit from BioAssay Systems (Hayward, CA) following the manufacturer’s protocol as previously utilized by us [18]. The kit is based on Jaffe method which quantifies picrate that forms a red colored complex with creatinine. The intensity of the color, measured at 510 nm, is directly proportional to creatinine concentration.

#### L-Carnitine

EnzyChromTM L-Carnitine Assay Kit (ELCR-100) from BioAssay Systems (Hayward, CA) was utilized to measure Carnitine concentration in urine samples following the manufacturer’s protocol. In this assay, a multistep reaction produces H_2_O_2_ which reacts with a specific dye to form a pink colored product. The optical density at 570 nm is directly proportional to the L-Carnitine concentration in the sample.

#### Pyruvate and lactate

Urinary pyruvate and lactate concentration was measured utilizing Pyruvate Assay Kit (Colorimetric) and L-lactate Colorimetric Assay Kit (Lactate Oxidase Method), respectively from RayBiotech Inc. (Peachtree Corners, GA) following manufacturer’s instructions. In pyruvate assay, pyruvate oxidase catalyzes the conversion of pyruvate to acetyl-phosphate, hydrogen peroxide (H_2_O_2_), and carbon dioxide. In lactate assay, lactate oxidase catalyzes the oxidation of L-lactate to pyruvate and hydrogen peroxide (H_2_O_2_). Peroxidase then catalyzes the reaction of hydrogen peroxide with the colorimetric probe to form a pink colored product. The OD measured at 520 nm is directly proportional to the concentration of pyruvate or lactate present in the sample.

#### α-ketoglutarate (α-KG)

Quantification of α-KG was performed in the urine samples utilizing α-KG assay kit (colorimetric) from Cayman Chemical (Ann Arbor, MI, USA) following manufacturers protocol. In this assay, alanine transaminase deaminates α-KG to form pyruvate. Pyruvate is then oxidized by pyruvate oxidase to yield acetyl phosphate, H_2_O_2_, and CO_2_ and downstream reaction produces a fluorescent compound resorufin detected at 570 nm, which is directly proportional to the α-KG concentration.

#### β-hydroxybutyrate (β-HB, ketone body)

Quantification of β-HB was performed in the urine samples utilizing β-HB assay kit (colorimetric) from Cayman Chemical (Ann Arbor, MI, USA) following manufacturers protocol. It is based upon the oxidation of D-3-hydroxybutyrate to acetoacetate by the enzyme 3-hydroxybutyrate dehydrogenase and cofactor NAD + is reduced to NADH. NADH reacts with the colorimetric detector WST-1 to produce a formazan dye with an absorbance maximum at 445–455 nm, which is directly proportional to the β-HB concentration.

### Statistical analysis for renal metabolomics results

Renal metabolomic values were log10-transformed to reduce distributional skewness. Multivariate data analysis was performed using both unsupervised Principal Component Analysis (PCA) and supervised Partial Least Squares Discriminant Analysis (PLS-DA) to explore the data structure and identify variables contributing to group differences. PCA was first applied as an unsupervised method for initial data overview and outlier detection, reducing variable count to key principle components (PCs) that explain maximum variance without using group labels. PLS-DA, a supervised classification technique, was subsequently employed to maximize separation between predefined experimental groups. This method identifies specific variables crucial for discrimination by maximizing the covariance between predictors and class response variables. For visualization of the metabolomics data, PLS-DA VIP scores plots were generated using MetaboAnalyst 6.0, a web based comprehensive metabolomics analysis tool [24]. Pathway enrichment analysis was conducted using MetaboAnalyst 6.0 integrated with the Small Molecule Pathway Database (SMPDB/Mus Musculus). Statistically significant differences in metabolites between biological sex groups (female vs. male) or age groups (4, 12, and 24 months) were assessed using the Wilcoxon rank-sum test. Metabolites with p-values < 0.05 and fold-change > 1.5 or < 0.667 were considered statistically significant and were used for pathway enrichment analysis. Multiple-testing correction was applied to the p-values from the Wilcoxon rank-sum tests using the Benjamini–Hochberg false discovery rate (FDR).

### Statistical analysis for other experiments

All values are expressed as mean ± SEM and results were analyzed using the statistical package from GraphPad Prism 7 (GraphPadSoftware Inc., La Jolla, CA). Statistical analysis was performed using two-way ANOVA to analyze the effect of age and sex, as well as interaction of age and sex (P_age*sex_). Tukey’s multiple comparisons post-hoc test was applied to examine the effect of age within each sex and impact of sex within same age groups and results are presented as impact of age (asterisks, *) and sex (hash, #) on the graph. The interaction of age and sex (P_age*sex_) are reported within each graph. P ≤ 0.05 was accepted as statistically significant.

## Results

### Age-dependent renal metabolic profiles in female mice at 4, 12, and 24 months

**Fig. 1:**
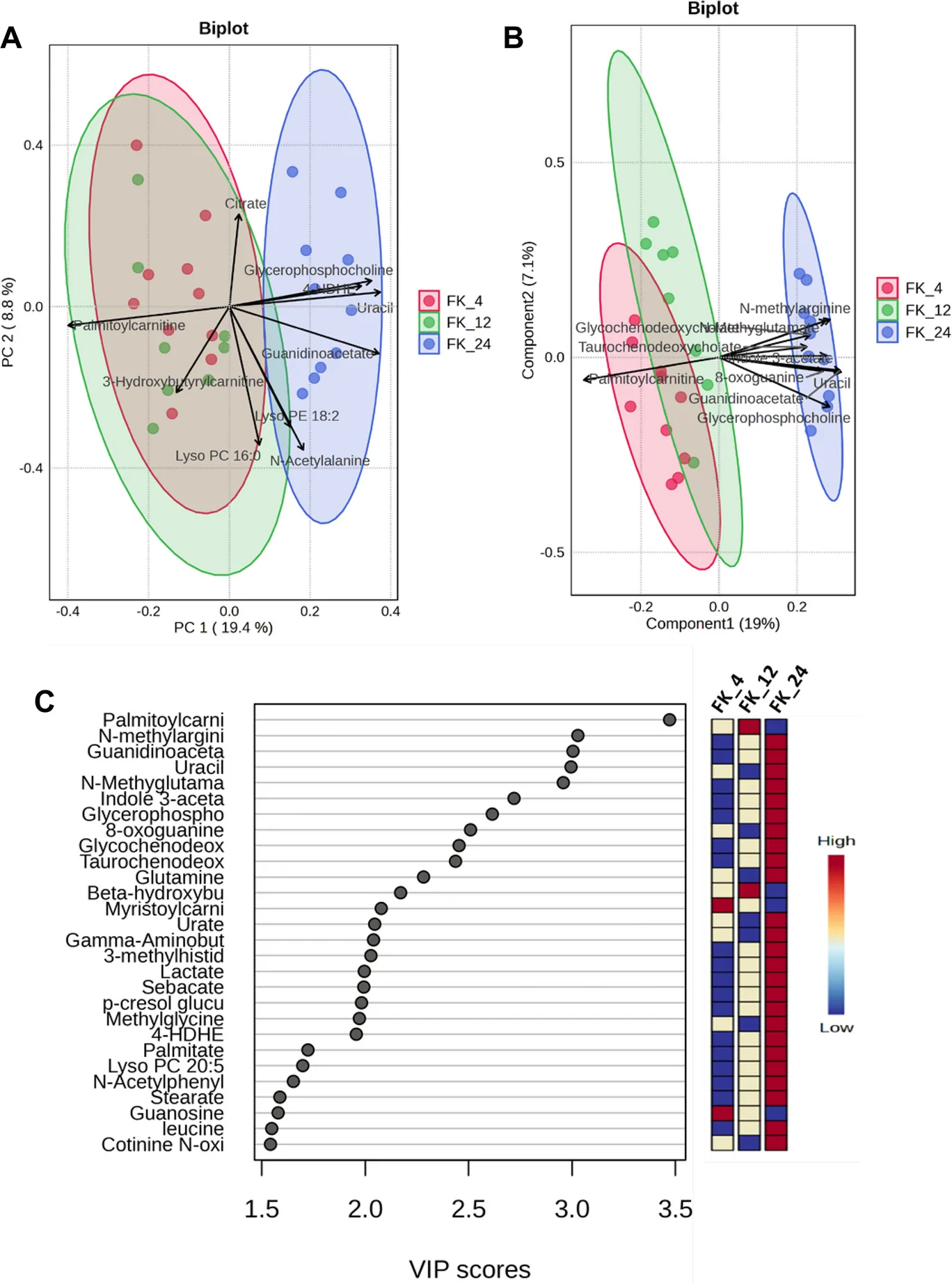
The renal metabolic landscape of the female mouse at 4, 12, and 24 months. (A) Principal Component Analysis (PCA) biplot illustrating the metabolic distribution across different age groups in female mouse kidneys. (B) Partial Least Squares Discriminant Analysis (PLS-DA) biplot highlighting age-associated separation in the renal metabolic profiles of female mice. (C) Variable Importance in Projection (VIP) score plot from the PLS-DA model, displaying metabolites with VIP scores > 1.5 that strongly contribute to group separation indicating their strong contribution to group separation. The heatmap panels on the right depict the average relative abundances of each metabolite in 4-, 12-, and 24-month female kidneys. Colors range from red (highest abundance) to blue (lowest abundance), illustrating age-dependent shifts in metabolite profiles. FK, female kidney

To investigate the influence of age on renal metabolic profiles in female mice, multivariate statistical analyses were performed using untargeted metabolomic data from 4-, 12-, and 24-month (M)-old animals. Principal Component Analysis (PCA) revealed age-dependent clustering of kidney samples (24M separated from 4 and 12M), suggesting substantial metabolic divergence at older age (Fig. 1A). The separation along the first two principal components captured 28.15 of the variances (PC1 = 19.37 and PC2 = 8.78) in the dataset indicates a progressive shift in metabolic phenotype with aging. Partial Least Squares Discriminant Analysis (PLS-DA) biplot further corroborated the distinct metabolic signatures associated with each age group (Fig. 1B). The model demonstrated (PC1 = 19, and PC2 = 7.1) a clear separation of 24M data set from 4 and 12M, with partial overlap between young and middle-aged kidneys, supporting the presence of robust and distinguishable age-related metabolic remodeling in the female mice kidneys. To identify specific metabolites contributing most significantly to group separation, a Variable Importance in Projection (VIP) score plot was generated from the PLS-DA model. Metabolites with VIP scores greater than 1.5 were considered key discriminators of age, which included several amino acid derivatives and fatty acid oxidation (FAO) intermediates (Fig. 1C) and supported a shift toward amino acid catabolism and reduced oxidative energy production in 24M mice kidney.

**Fig. 2:**
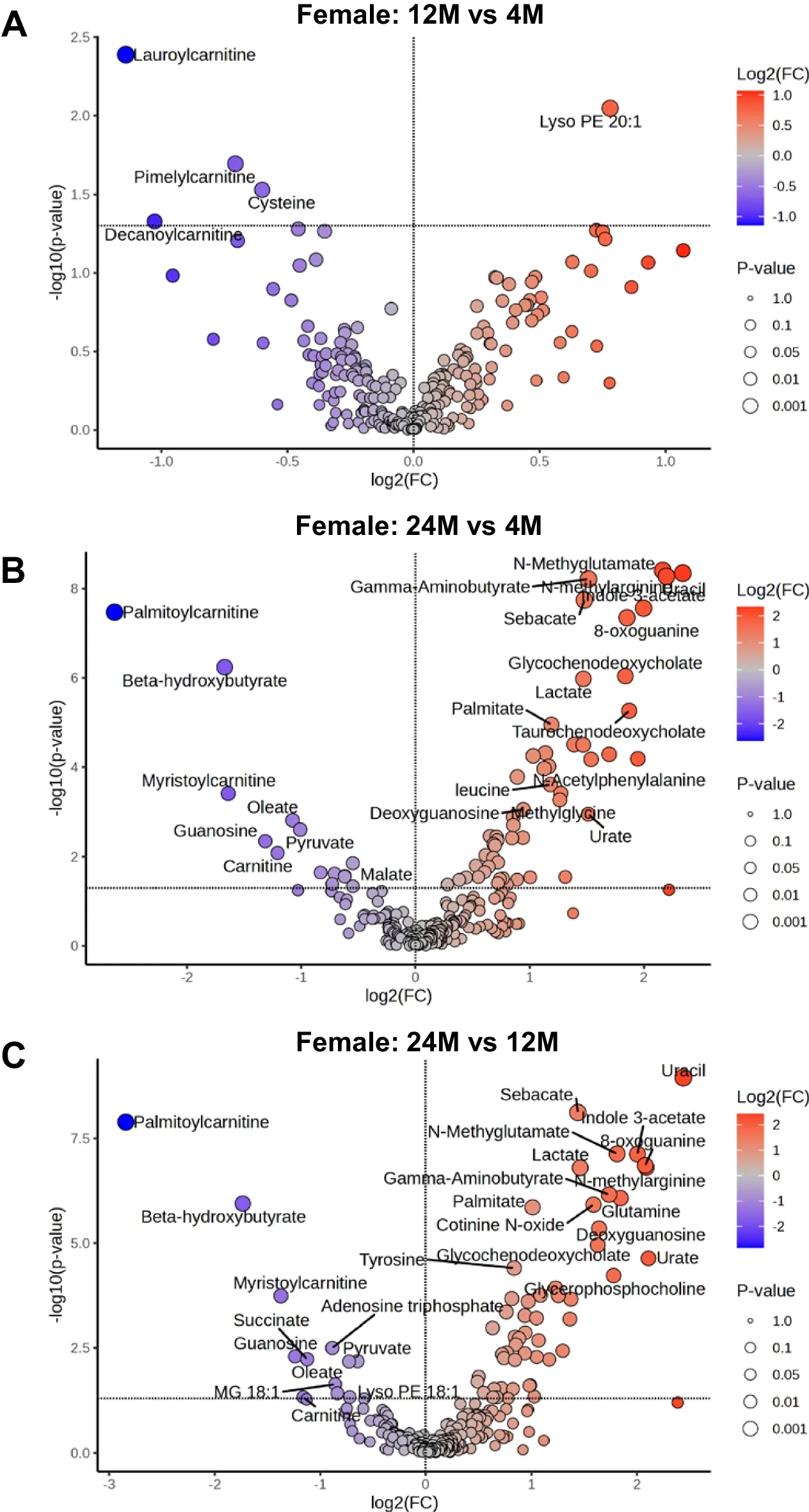
Renal metabolic profiles of female mice across age groups (4, 12, and 24 months). (A) Volcano plot depicting significantly altered (p < 0.05) renal metabolites between 12 and 4M female mice. B) Volcano plot illustrating differential renal metabolites between 24 and 4M female mice. C) Volcano plot showing metabolite changes between 24 and 12M female mice

Further, univariate analysis of the renal metabolome data of female mice showed considerable interplay between renal metabolism and epigenetics in age-related alterations. Females showed increased levels of a single metabolite (Lyso PE 20:1) and decreased levels of four metabolites lauroylcarnitine, pimelylcarnitine, decanoylcarnitine, and cysteine at 12M compared to 4M female mice (Fig. 2A and Supplementary Table 1). These findings suggest a potential impairment in renal FAO intermediates as indicated by the reduced levels of fatty acyl carnitines. Comparison between 24 and 4M female mice revealed a broad and significant metabolic reprogramming in the kidney associated with aging. A total of fifty-three metabolites were present in significantly higher abundance with a strong enrichment in amino acid derivatives, while fourteen metabolites were deregulated, primarily those involved in FAO and energy metabolism (Fig. 2B and Supplementary Table 2). The marked reduction in FAO metabolites—including β-hydroxybutyrate (β-HB), various fatty acylcarnitines, and tricarboxylic acid cycle (TCA) intermediates such as pyruvate, citrate, and ATP—suggests a significant decline in mitochondrial oxidative capacity in aged female kidneys. At the same time, the accumulation of amino acid-related metabolites, particularly argininosuccinate, aspartate, oxoglutarate, and glutamine, points to potential dysregulation in nitrogen handling, including urea cycle and ammonia detoxification pathways. Furthermore, the elevation of 8-oxoguanine (8-oxoG), a marker of oxidative DNA damage, highlights the presence of increased oxidative stress in the aging kidney. Comparison between 24 and 12M female mice revealed a total of fifty-one metabolites were significantly elevated in the 24M group, many of which are involved in branched-chain amino acid (BCAA) degradation (e.g., leucine, isoleucine, valine) and nitrogen waste processing, including components of the urea and ammonia cycles. Conversely, twelve metabolites were reduced in the older group, predominantly those associated with FAO, such as β-HB, palmitoylcarnitine, and myristoylcarnitine (Fig. 2C and Supplementary Table 3).

Together, these findings highlight a metabolic remodeling in the aging female kidney, characterized by reduced lipid utilization and increased amino acid degradation, increased oxidative stress, and dysregulated nitrogen recycling which may serve as both a marker and a mechanistic contributor to renal aging. A representative visualization of the metabolic pathway enrichment analysis in female kidneys (24 vs 4 months) is presented in Supplementary Fig. 1. A heatmap illustrating the relative abundances of significantly altered metabolites among female kidneys across three age groups is provided in Supplementary Fig. 2. Adjusted p-values of statistically significant metabolites from the Wilcoxon rank-sum tests comparing 12M vs 4M, 24M vs 4M, and 24M vs 12M in female kidney samples are presented in Supplementary Tables 1, 2, and 3, respectively.

### Age-dependent renal metabolic profiles in male mice at 4, 12, and 24 months

**Fig. 3:**
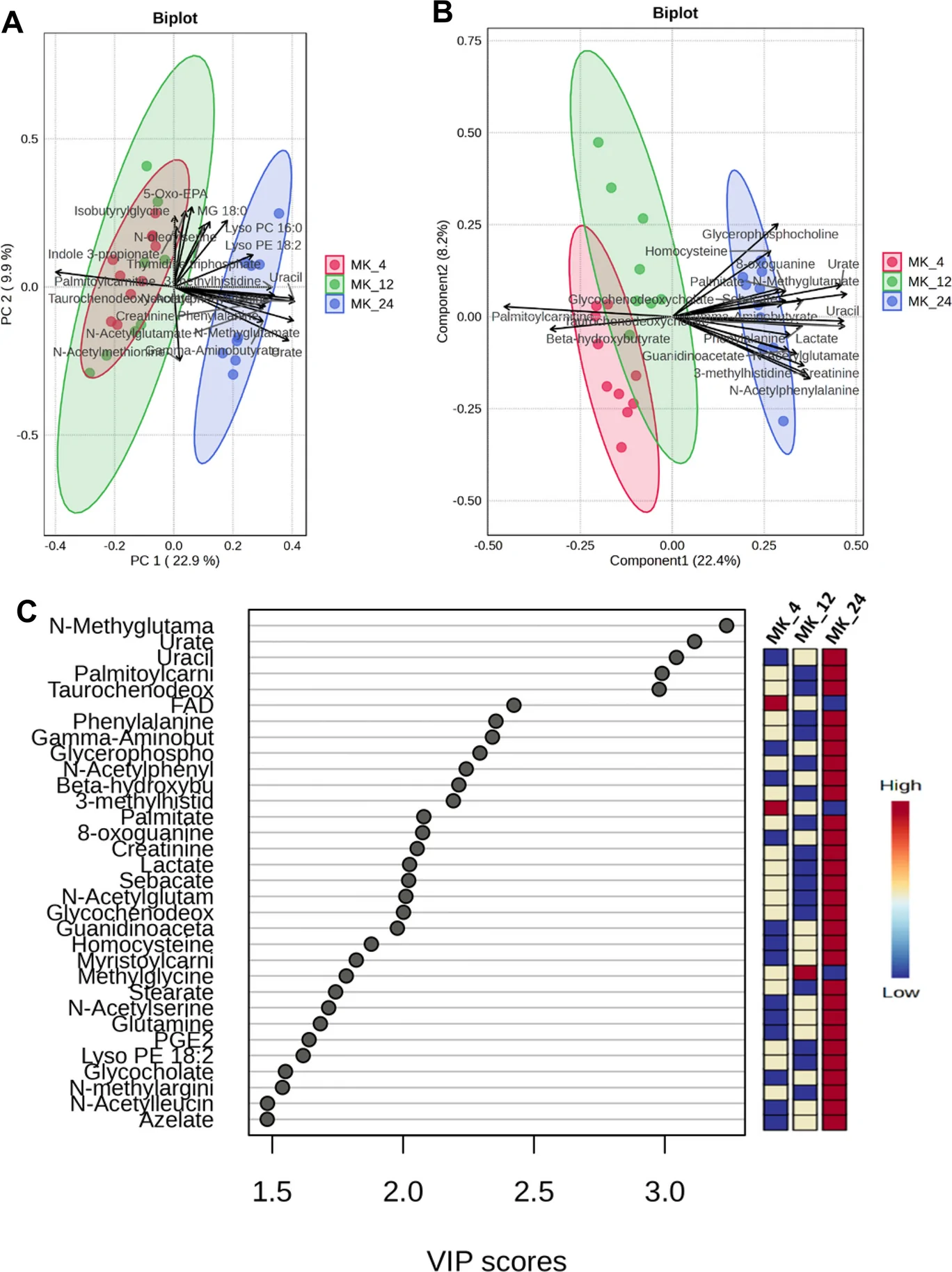
The renal metabolic landscape of the male mouse at 4, 12, and 24 months. (**A**) Principal Component Analysis (PCA) biplot illustrating the metabolic distribution across different age groups in male mouse kidneys. (**B**) Partial Least Squares Discriminant Analysis (PLS-DA) biplot highlighting age-associated separation in the renal metabolic profiles of male mice. (**C**) Variable Importance in Projection (VIP) score plot from the PLS-DA model, displaying metabolites with VIP scores > 1.5 that strongly contribute to group separation. The heatmap panels on the right depict the average relative abundances of each metabolite in 4-, 12-, and 24-month male kidneys. Colors range from red (highest abundance) to blue (lowest abundance), illustrating age-dependent shifts in metabolite profiles. MK, male kidney

The PCA analysis of the renal metabolome in 4M, 12M, and 24M-old male mice revealed distinct age-related clustering, with the 24M group clearly separated from the 4M and 12M groups (Fig. 3A). The first two principal components accounted for 32.75% of the total variance, indicating substantial age-associated remodeling of the renal metabolic landscape. The clear spatial separation between the youngest and oldest groups underscores the progressive nature of metabolic reprogramming in the aging male kidney. The PLS-DA biplot provided further resolution of age-specific differences, displaying a clearer separation among all three age groups with partial overlap between young and middle-aged mice (Fig. 3B). The VIP score plot derived from the PLS-DA model identified key metabolites contributing most significantly to group discrimination (Fig. 3C). Metabolites with VIP scores greater than 1.5 included amino acid derivatives, N-acetylated compounds, and intermediates of lipid metabolism, consistent with our broader findings of increased amino acid catabolism, oxidative stress, and inflammatory lipid signaling in aged male kidneys.

Collectively, the distinct trajectory revealed through PCA and PLS-DA, along with the identification of key VIP-associated metabolites, underscores a robust biological response to aging that likely contributes to renal functional decline and increased susceptibility to metabolic comorbidities in males.

**Fig. 4:**
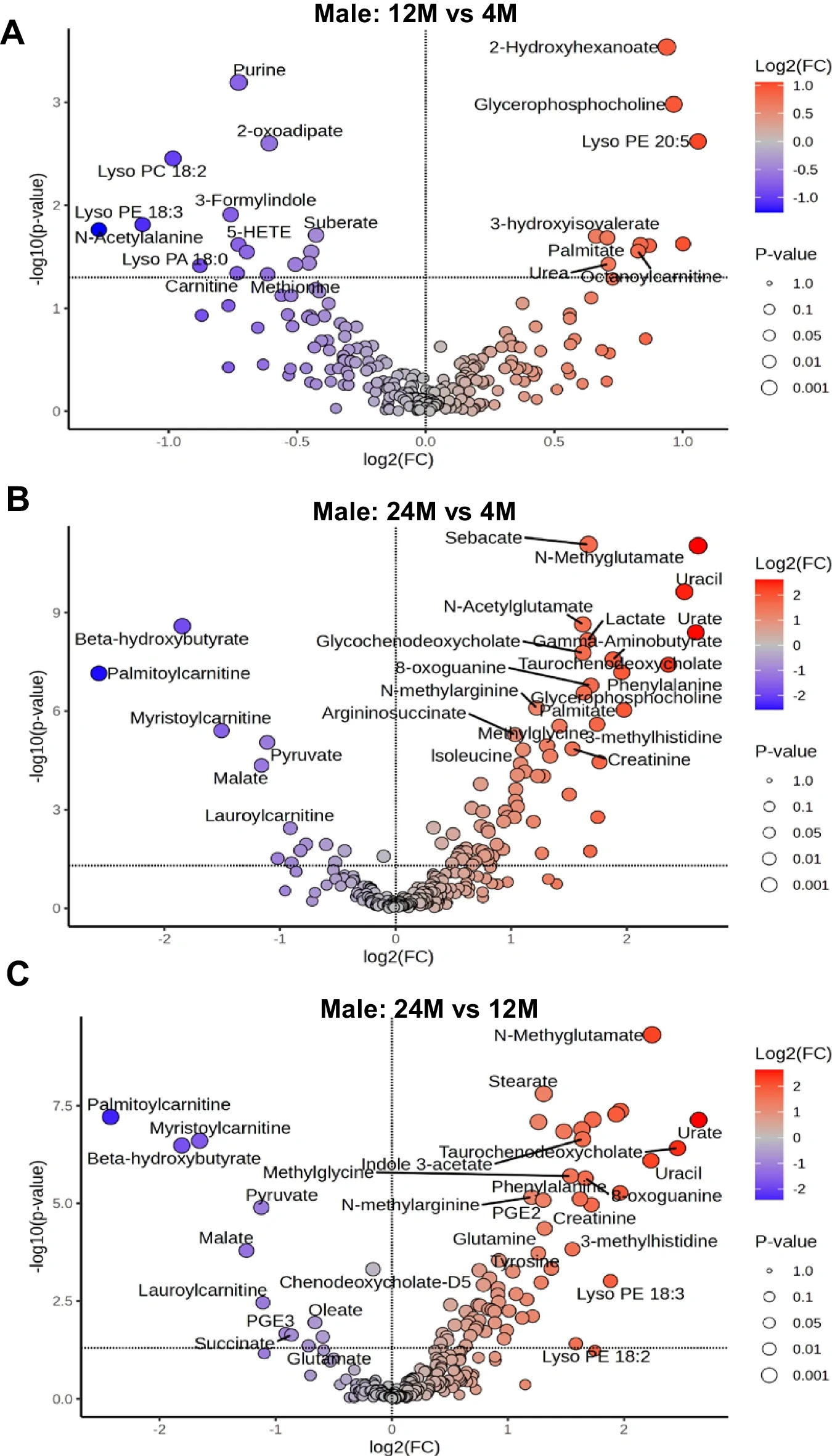
Renal metabolic profiles of male mice across age groups (4, 12, and 24 months). (**A**) Volcano plot depicting significantly altered (p < 0.05) renal metabolites between 12 and 4M male mice. (**B**) Volcano plot illustrating differential renal metabolites between 24- and 4-month-old male mice. (**C**) Volcano plot showing metabolite changes between 24 and 12M male mice

Further, univariate analysis of the renal metabolome data of male mice revealed significant age-dependent shifts, highlighting progressive biochemical remodeling across the lifespan. Ten metabolites were upregulated, primarily comprising lipids (e.g., *Lyso PE 20:5*, *Lyso PC 18:1*, *Lyso PA 18:1*) and fatty acids (*2-hydroxyhexanoate*, *palmitate*, *3-hydroxyisovalerate*) in 12M old mice compared to 4M old male mice. Fifteen metabolites were downregulated, including amino acid derivatives (*N-acetylaspartylglutamate*, *5-methylcytosine*, *N-acetylalanine*) and lysophospholipids (*Lyso PC 20:4*, *Lyso PA 18:0*, *Lyso PC 18:2*, *Lyso PE 18:3*) (Fig. 4A & Supplementary Table 4), suggesting reduced amino acid metabolism and phospholipid diversity, potentially reflecting diminished biosynthetic activity. A total of sixty-eight metabolites were significantly elevated, primarily linked to amino acid metabolism in 24M mice compared with 4M mice. These included N-acetylated and methylated amino acids such as *N-acetylphenylalanine*, *3-methylhistidine*, *N-acetylglutamate*, *methylglycine*, *N-acetylserine*, and *N-methylarginine*. This accumulation indicates enhanced amino acid catabolism, post-translational modifications, and potential epigenetic regulation—processes associated with cellular aging, oxidative stress, and chronic low-grade inflammation. These metabolic signatures may represent compensatory mechanisms in response to proteostasis imbalance and mitochondrial dysfunction. Conversely, fourteen metabolites were downregulated, including key components of FAO, such as multiple acylcarnitines and β-HB, suggesting an age-related decline in mitochondrial lipid oxidation (Fig. 4B and Supplementary Table 5). Further comparison between 12 and 24M males revealed sixty-three metabolites were upregulated in 24M kidneys, notably in pathways related to urea cycle activity, with elevated *argininosuccinate*, *alanine*, *oxoglutarate*, and *glutamine*, indicate increased nitrogen handling and amino acid turnover. Inflammatory oxylipin metabolism was elevated, marked by *LTB4*, *PGE2*, *14,15-DiHETrE*, *THB2*, and *5-HETE*, reflecting enhanced inflammatory signaling and lipid mediator production- hallmarks of immunosenescence and chronic kidney injury. Glycine/serine metabolism was altered, with elevated *guanidinoacetate*, *sarcosine*, *oxoglutarate*, and *alanine* levels, indicate altered one-carbon metabolism and redox regulation. In contrast, eleven metabolites were significantly reduced, including central intermediates of glycolysis and the TCA cycle (*glutamate*, *malate*, *pyruvate*, *succinate*), as well as guanosine and GTP (Fig. 4C and Supplementary Table 6). These changes may reflect a general decline in central carbon metabolism, pointing to diminished bioenergetic capacity and anabolic activity in aging kidneys.

Collectively, these findings illustrate a clear metabolic shift in aging male kidneys, characterized by early lipid remodeling and amino acid decline by 12 months; a pronounced rise in amino acid catabolism and inflammatory lipid mediators along with a progressive impairment of mitochondrial FAO and central energy metabolism by 24 months. A representative visualization of the metabolic pathway enrichment analysis in male kidneys (24 vs 4 months) is presented in Supplementary Fig. 3. A heatmap illustrating the relative abundances of significantly altered metabolites among male kidneys across three age groups is provided in Supplementary Fig. 4. Adjusted p-values of statistically significant metabolites from the Wilcoxon rank-sum tests comparing 12M vs 4M, 24M vs 4M, and 24M vs 12M in male kidney samples are presented in Supplementary Tables 4, 5, and 6, respectively.

### Biological sex and age-associated clustering of renal metabolites/pathways in mice

**Fig. 5:**
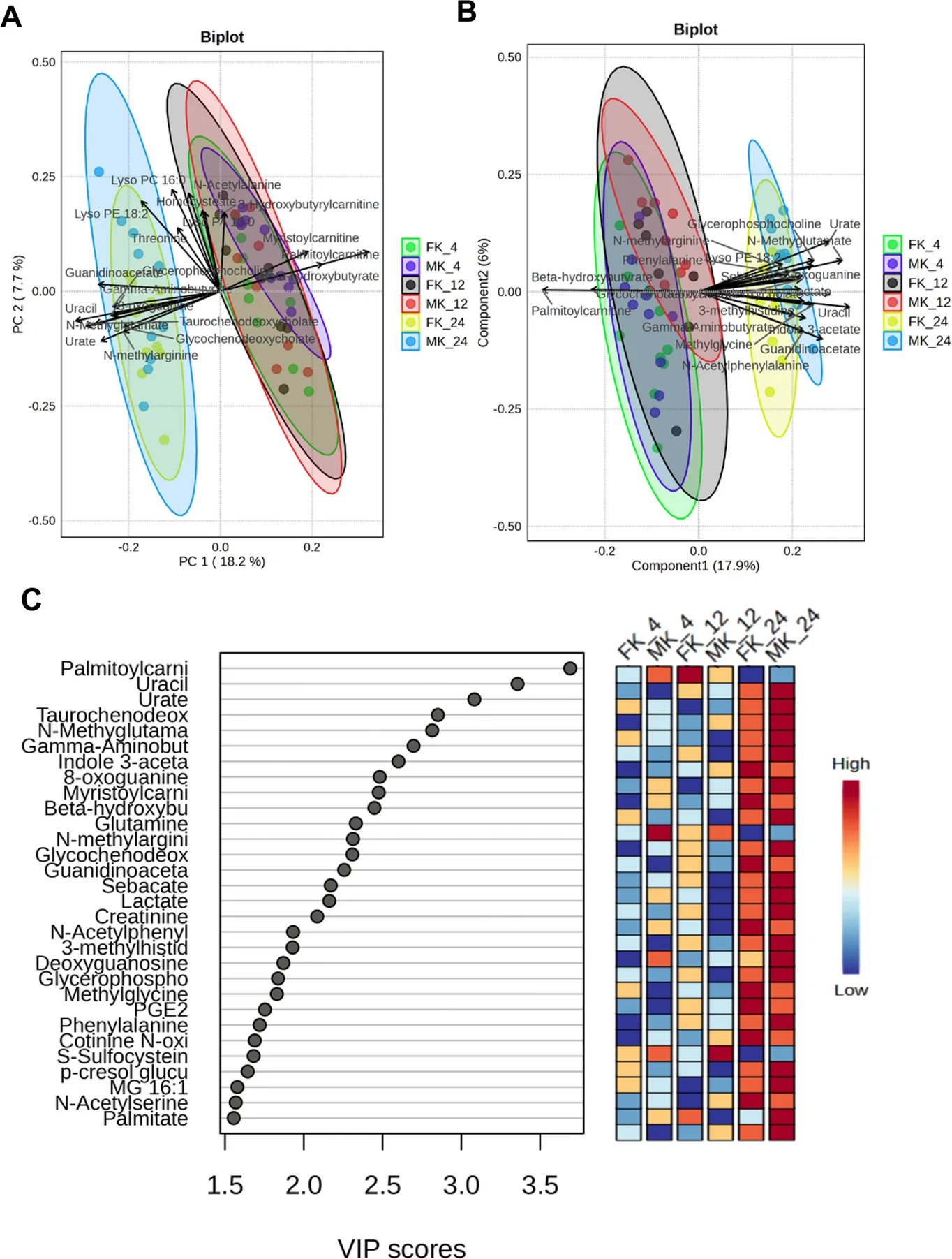
The renal metabolic landscape comparing between female and male mouse at 4, 12, and 24 months. (**A**) Principal Component Analysis (PCA) biplot illustrating the metabolic distribution across different age groups in male mouse kidneys. (**B**) Partial Least Squares Discriminant Analysis (PLS-DA) biplot highlighting age-associated separation in the renal metabolic profiles of male mice. (**C)** Variable Importance in Projection (VIP) scores from the PLS-DA model, illustrating metabolites with VIP scores > 1.5 that strongly contribute to group separation. The heatmap panels on the right show the average relative abundances of each metabolite in 4-, 12-, and 24-month female and male kidneys. Colors range from red (highest abundance) to blue (lowest abundance), highlighting age-dependent shifts in metabolite profiles across both sexes. FK, female kidney; MK, male kidney

To investigate how biological sex modulates renal metabolic profiles across the lifespan, multivariate analyses were conducted on metabolomic data sets from male and female mice at 4-, 12-, and 24- months of age. The PCA revealed a close cluster by sex, while a partial distinct clustering by age, underscoring the combined influence of these factors on the renal metabolome. At all age groups male and female kidneys clustered closely (Fig. 5A). The PLS-DA biplot confirmed and extended the PCA findings by demonstrating robust stratification of all six experimental groups (Fig. 5B). Each sex-age group formed a partial discrete cluster and the separation between the 24M male and female groups was particularly pronounced. The intermediate 12M clusters for each sex showed partial overlap with their younger and older counterparts, suggesting transitional metabolic states. Variable Importance in Projection analysis identified key metabolites driving this sex- and age-dependent stratification (Fig. 5C). Metabolites with VIP scores greater than 1.5 included a variety of amino acid derivatives, urea cycle intermediates, lysophospholipids, and acylcarnitines. Notably, several sex-specific markers emerged: aged females exhibited elevated levels of amino acids such as leucine, glutamine, and serine, phosphoserine, indolacetylglutamine, and N-methylarginine while aged males showed increased levels of inflammatory oxylipins and lipid signaling molecules. Reduced acylcarnitines in aged females pointed to diminished mitochondrial FAO, whereas elevated urea cycle metabolites in males implied enhanced nitrogen disposal and amino acid turnover. These differential metabolic signatures provide further evidence for sex-specific vulnerabilities in renal aging—namely, bioenergetic decline in females versus inflammatory remodeling in males.

In summary, the distinct clustering observed in PCA and PLS-DA, along with the discriminatory metabolites highlighted in the VIP analysis, demonstrate that the renal metabolome evolves in a sex-specific manner at older age.

**Fig. 6:**
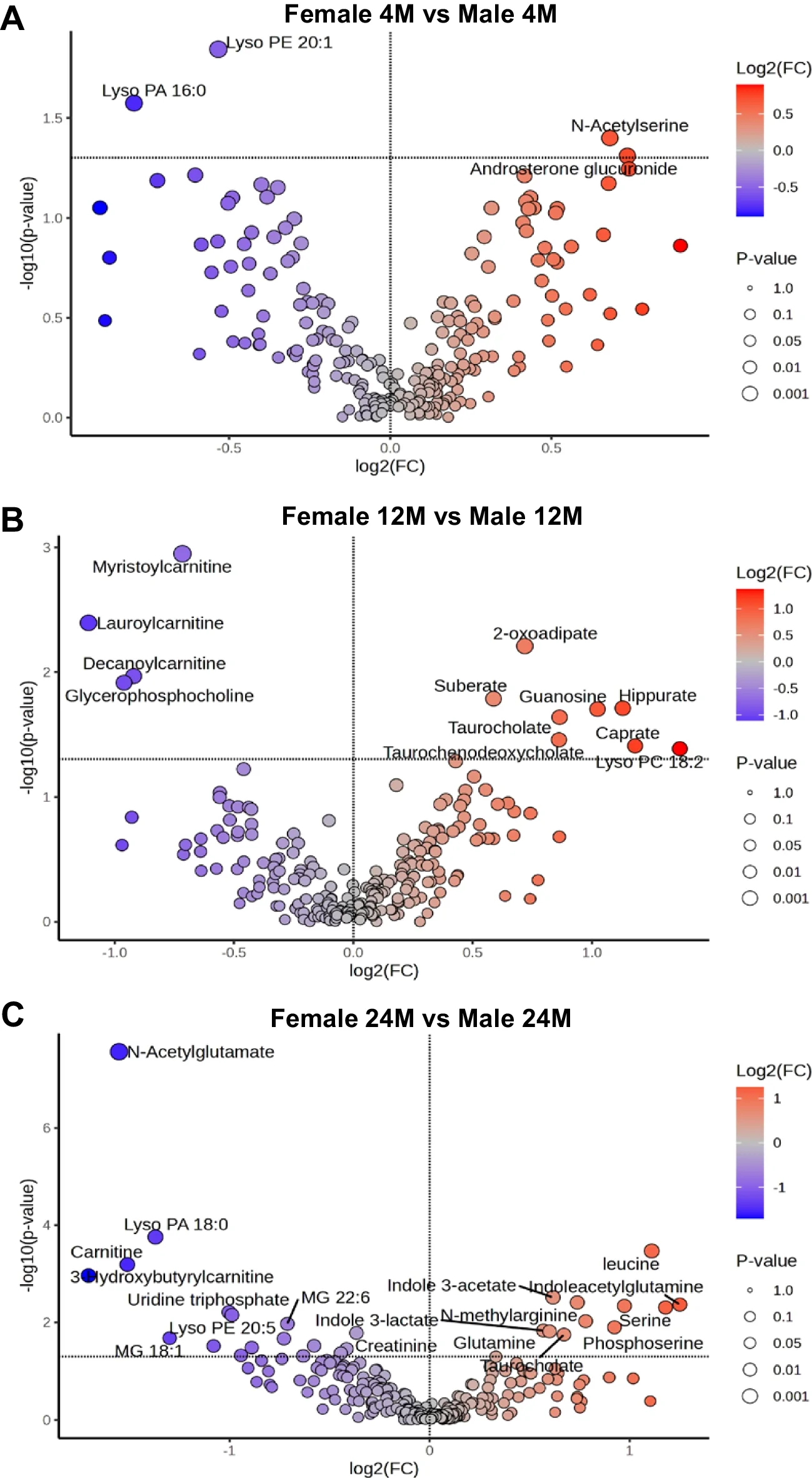
Sex associated renal metabolic profiles in mice at 4, 12, and 24 months. Volcano plots illustrating sex-associated differences in renal metabolite levels across different ages in mice. Panels (**A**), (**B**), and (**C**) depict the distribution of differentially expressed metabolites between female and male mice at 4M, 12M, and 24M of age, respectively. Metabolites with p-values < 0.05 are highlighted, indicating significant sex-based metabolic differences at each age

Further in univariate analysis, sex-specific differences in kidney metabolite levels were evident across all age groups. In 4M mice, females showed two upregulated metabolites—N-acetylserine and androsterone glucuronide and two downregulated metabolites—Lyso PE 20:1 and Lyso PA 16:0 compared to age-matched males (Fig. 6A and Supplementary Table 7). The altered levels of lysophospholipids may suggest early divergence in membrane lipid remodeling or phospholipid turnover between sexes. These differences became more evident with age, in 12M mice, females showed eight significantly upregulated metabolites, including bile acids, purine/pyrimidine derivatives, and fatty acid metabolites, while four metabolites, primarily fatty acylcarnitines were downregulated compared to males (Fig. 6B and Supplementary Table 8). The most pronounced sex differences were observed in aged mice, as 24M females exhibited eleven significantly elevated metabolites—mostly amino acids such as leucine, serine, glutamine, phosphoserine, indolacetylglutamine, and N-methylarginine and seventeen reduced metabolites, the majority of which were lysophospholipids (including monoacylglycerols, Lyso PE, and Lyso PA), as well as creatinine and carnitine, compared to age-matched males (Fig. 6C and Supplementary Table 9). These changes likely reflect cumulative effects of aging on renal function, metabolic demand, and hormonal regulation. Adjusted p-values of statistically significant metabolites from the Wilcoxon rank-sum tests comparing female vs male at 4M, 12M, and 24M are presented in Supplementary Tables 7, 8, and 9, respectively.

In summary, these findings underscore the progressive emergence and intensification of sex-based metabolic differences in the kidney across the lifespan between males and females. A heatmap illustrating the relative abundances of significantly altered metabolites among female, and male kidneys across three age groups is provided in Supplementary Fig. 5.

Metabolites showing significant associations with age, sex, or their interaction were identified using a two-way ANOVA with sex and age group (4, 12, and 24 months) as fixed factors. Multiple-testing correction was applied using the Benjamini–Hochberg false discovery rate (FDR), and metabolites with q < 0.05 were considered statistically significant. The overlap among metabolites affected by age, sex, and the age × sex interaction is illustrated as a Venn diagram in Supplementary Fig. 6. The corresponding lists of overlapping metabolites are provided in Supplementary Table 10.

### Biological sex- and age-associated changes in metabolic enzymes

**Fig. 7:**
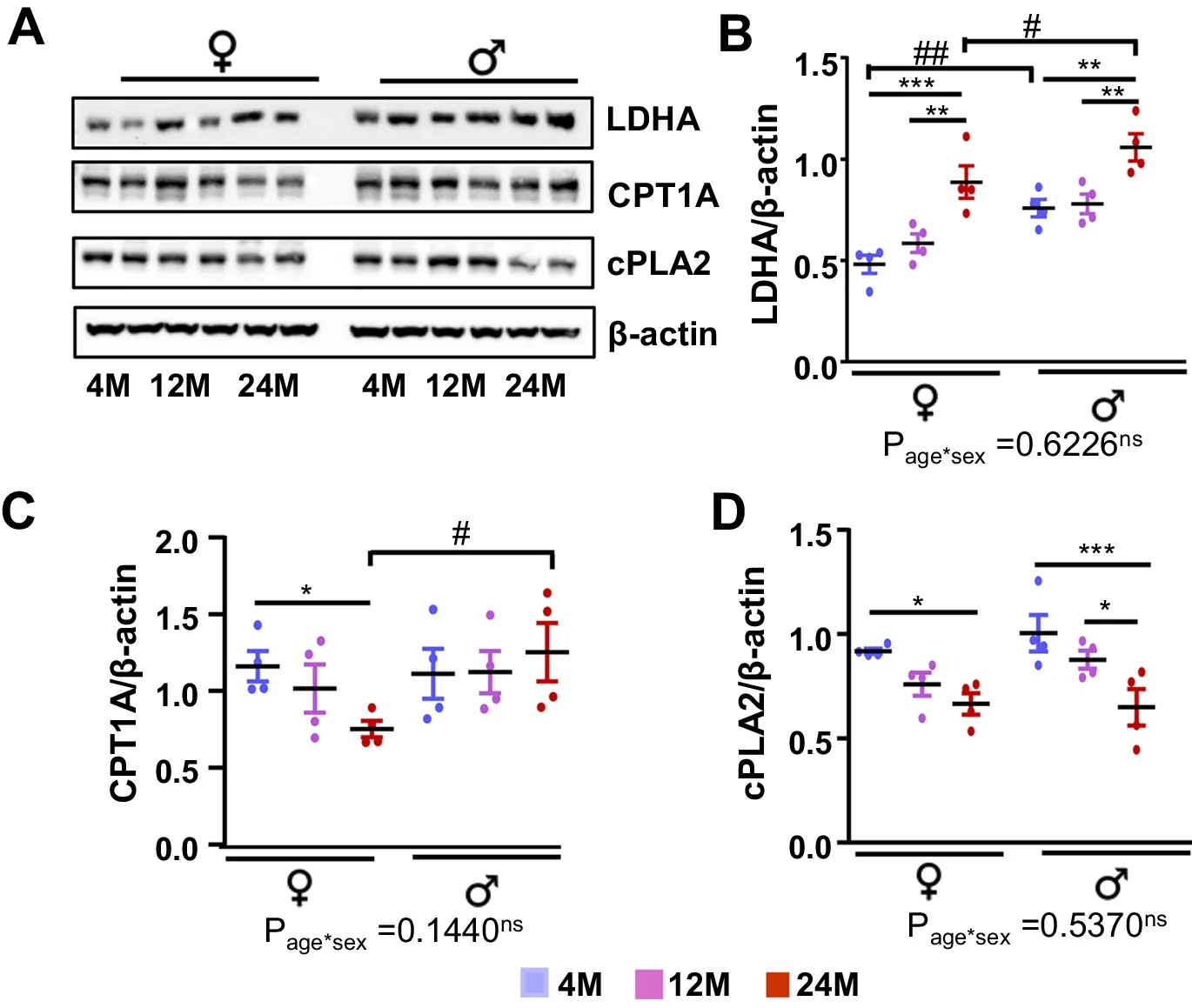
Renal expression of metabolic enzymes associated with sex and age in mice kidney. (**A-D**) Western blot and densitometric analyses of LDHA, CPT1A, and cPLA2 in the renal extract prepared from 4M, 12M, and 24M mice kidney tissues. β-actin was used as loading control (n = 8). Each dot represents results from 2 mice kidney tissues. Two-way ANOVA with Tukey’s multiple comparison test was performed to study the effect of age within same sex (*p < 0.05, **p < 0.01, ***p < 0.001) and effect of sex in age-matched groups (#p < 0.05, ##p < 0.01) and the interaction of age and sex (P_age*sex_) are presented in the graph. N = 8 (each dot represents sample from 2 mice); ns, non-significant; LDHA, lactate dehydrogenase A; CPT1A, carnitine palmitoyltransferase 1 A; cPLA2, cytosolic phospholipase A2

To further validate the aging-associated increase in lactate and decrease in pyruvate levels observed in both sexes, we assessed the protein expression of lactate dehydrogenase A (LDHA), a key glycolytic enzyme. During anaerobic glycolysis LDHA catalyzes the conversion of pyruvate to lactate and is widely recognized as a potential marker of tissue injury. Western blot and densitometry analysis revealed a significant upregulation of LDHA protein levels in 24M male and female mice (Fig. 7, A and B), consistent with enhanced glycolytic flux and potential metabolic stress in aging kidneys. Notably, a sex-specific difference was observed in 4M mice, where female mice exhibited significantly lower LDHA levels compared to age-matched males. Significantly reduced carnitine levels were observed in 24M females compared to age-matched males, henceforth, we determined protein levels of carnitine palmitoyltransferase 1 A (CPT1A), a rate-limiting enzyme in mitochondrial long-chain FAO. Correspondingly CPT1A protein levels were significantly decreased in 24M females compared to 4M females and 24M males (Fig. 7, A and C). No significant age-related changes in CPT1A expression were observed in males, further emphasizing sex-specific regulation of mitochondrial fatty acid metabolism during aging in females. Additionally, we examined cytosolic phospholipase A2 (cPLA2), a key enzyme involved in membrane phospholipid turnover and lipid signaling. While increased cPLA2 activity has been implicated in kidney injury and inflammation, we found a significant age-related decrease in cPLA2 protein levels in both sexes (Fig. 7, A and D). This reduction may reflect a compensatory response to limit pro-inflammatory lipid mediator production in the aging kidney, though the functional consequences remain to be fully elucidated.

### Age-associated regulation of renal metabolite cofactors essential for epigenetic enzymes involved in histone acetylation/deacetylation modifications

**Fig. 8:**
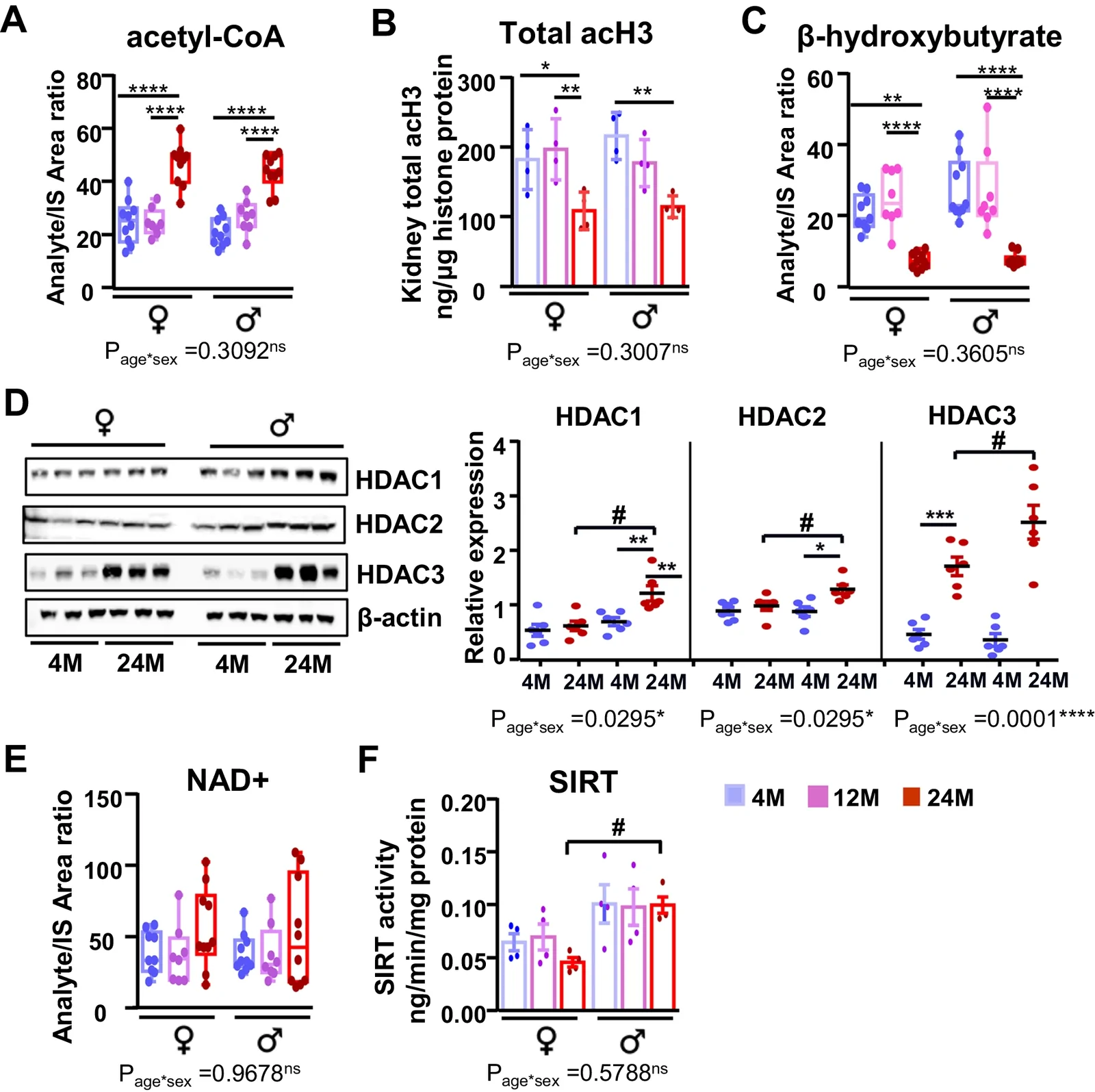
Age- and sex-associated regulation of renal metabolite cofactors essential for histone acetylation/deacetylation in mice kidneys. (**A**) analyte/IS area ratio of acetyl-CoA in mice renal tissues. (**B**) Total H3 acetylation levels in the renal histones extracted from kidney tissues; n = 8 (each dot represents 2 samples) (**C**) analyte/IS area ratio of β-HB in mice renal tissues (**D**) Western blots and densitometry analyses of class I HDACs, (HDAC1, HDAC2 and HDAC3) protein levels in renal extracts; n = 6. (**E**) analyte/IS area ratio of NAD^+^ in mice renal tissues and (**F**) Quantification of SIRT enzymatic activity in renal nuclear extracts; n = 8 (each dot represents 2 samples). Two-way ANOVA with Tukey’s multiple comparison test was performed to study the effect of age within same sex (*p < 0.05, **p < 0.01, ***p < 0.001 ****p < 0.0001) (age differences, same sex) and effect of sex in age-matched groups (#p < 0.05), and the interaction of age and sex (P_age*sex_) are presented in the graph. n = 8–10 (for analyte/IS area ratio results); ns, non-significant; HDAC, histone deacetylase; NAD, nicotinamide adenine dinucleotide; SIRT, sirtuins

Metabolites act as crucial cofactors for epigenetic enzymes and thereby play a fundamental role in regulating the epigenome. To evaluate this interplay during aging, we analyzed three TCA cycle–derived renal metabolite cofactors (ac-CoA, β-HB, and NAD⁺) essential for histone acetyltransferase (HAT) and histone deacetylase (HDAC) enzymatic activity, key regulators of histone acetylation and deacetylation, respectively. We observed significantly elevated levels of ac-CoA—a required cofactor for HAT activity, in 24M male and female mice compared to 4M and 12M sex-matched controls (Fig. 8A). Paradoxically, total acetylated histone H3 (acH3) levels were reduced in aged mice of both sexes (Fig. 8B). This finding aligns with our recent observations showing that HAT activity increases at 12M but declines significantly by 24M in both males and females [19] suggesting a decoupling between ac-CoA availability and HAT function in advanced age. Additionally, β-HB, an endogenous class I HDAC inhibitor, was markedly reduced in aged males and females compared to younger groups (Fig. 8C), indicating a potential loss of HDAC inhibition with age. Consistent with this, HDAC1 and HDAC2 protein levels were significantly upregulated in aged male mice, while HDAC3 was elevated in both sexes at 24M relative to younger counterparts (Fig. 8D), pointing to age-associated increases in HDAC-mediated deacetylation. In contrast, NAD⁺ levels, a critical cofactor for class III HDACs (sirtuins), remained stable across all age groups, and total sirtuin activity showed no significant age-related change in either sex (Fig. 8, E and F). However, sex-specific difference in sirtuin activity was evident, with significantly lower activity in aged females compared to aged males, suggesting sex-linked regulation of sirtuin function in the aging kidney.

### Age-associated regulation of renal metabolite cofactors essential for epigenetic enzymes involved in histone and DNA methylation/demethylation modifications

**Fig. 9:**
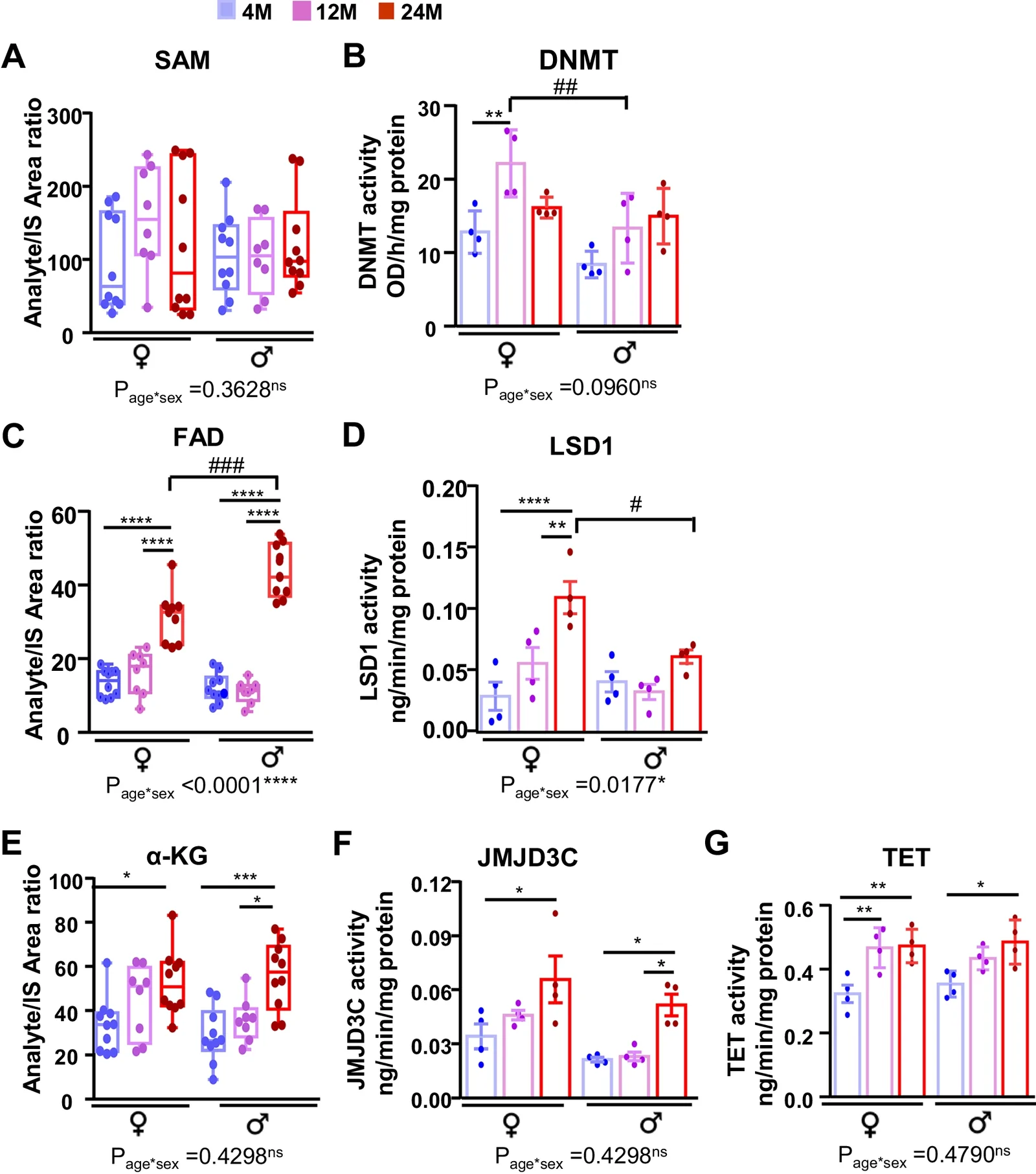
Age- and sex-associated regulation of renal metabolite cofactors essential for histone and DNA methylation/demethylation in mice kidneys. (**A**) analyte/IS area ratio of SAM in mice renal tissues, (**B**) quantification of DNMT enzymatic activity in renal nuclear extracts. (**C**) analyte/IS area ratio of FAD, (**D**) LSD1 enzymatic activity in renal nuclear extracts, (**E**) analyte/IS area ratio of α-KG in mice renal tissues and quantification of enzymatic activities of (**F**) JMJD3C and (**G**) TET enzymes in the renal nuclear extracts. Two-way ANOVA with Tukey’s multiple comparison test was performed to study the effect of age within same sex (*p < 0.05, **p < 0.01, ***p < 0.001 ****p < 0.0001) (age differences, same sex) and effect of sex in age-matched groups (#p < 0.05, ##p < 0.01, ####p < 0.0001), and the interaction of age and sex (P_age*sex_) are presented in the graph. n = 8–10 (for analyte/IS area ratio results; n = 8 (for epigenetic enzymes activity; each dot represents 2 samples); ns, non-significant. SAM, S-adenosyl methionine; DNMT, DNA methyltransferases; FAD, flavin adenine dinucleotide; LSD1, lysine-specific demethylase 1; αKG, α-ketoglutarate; JMJD3C, Jumonji C domain-containing histone demethylase protein 3; TET, ten-eleven translocation

We further examined renal metabolite cofactors essential to the enzymatic activity of histone and DNA methyltransferases (HMTs and DNMTs), as well as histone demethylases (HDMs) and DNA demethylases. S-adenosyl methionine (SAM), a key methyl donor utilized by both HMTs and DNMTs, exhibited comparable levels across all age groups and both sexes (Fig. 9A), indicating that substrate availability for methylation may not be a limiting factor during aging. Despite stable SAM levels, DNMT enzymatic activity was significantly elevated in 12M female mice compared to 4M females and 12M males, followed by a decline at 24M (Fig. 9B). This suggests that DNMT activity is modulated not solely by cofactor availability, but potentially by regulatory proteins or post-translational modifications. Renal levels of FAD, a cofactor for lysine-specific demethylase 1 (LSD1) activity, was significantly elevated in 24M females and males compared to both sex-matched younger age groups (4M and 12M) (Fig. 9C). Interestingly, aged male mice exhibited higher FAD levels than their female counterparts, highlighting sex-specific metabolic differences. However, LSD1 activity was significantly increased only in 24M female mice compared to younger females, despite males having higher FAD levels (Fig. 9D). This finding implies that factors beyond FAD availability such as LSD1 protein expression or post-translational modifications, may influence its activity. Levels of α-KG, a TCA cycle intermediate and essential cofactor for Jumonji C domain-containing histone demethylases (JMJDs), were significantly elevated in 24M mice of both sexes compared to 4M mice (Fig. 9E). Correspondingly, JMJD protein 3 (JMJD3C) activity was markedly increased in aged mice of both sexes relative to their younger counterparts (Fig. 9F), suggesting an age-related upregulation of histone demethylation capacity. Additionally, enzymatic activity of ten-eleven translocation (TET) enzymes, α-KG/Fe(II)–dependent dioxygenases involved in active DNA demethylation, was elevated in 12M and 24M females compared with 4M females, and in 24M males compared to both 4M and 12M males (Fig. 9G). These findings suggest an age- and sex-dependent enhancement of DNA demethylation pathways in the kidney.

Overall, these results indicate that while metabolite levels such as SAM, FAD, and α-KG fluctuate with age and sex, enzymatic activity does not always directly correlate with cofactor abundance. This highlights the importance of additional regulatory mechanisms, such as enzyme expression levels, post-translational modifications, and protein–protein interactions, in modulating epigenetic enzyme activity across the lifespan.

### Assessment of urinary metabolite profiles by age and sex in humans

Metabolite profiling of human biofluids offers a non-invasive snapshot of physiological and metabolic states. To evaluate age- and sex-associated metabolic changes, we analyzed key intermediates of the TCA cycle, lactate, pyruvate, and α-KG, as well as related metabolites L-carnitine and β-HB in urine samples from men and women at different ages. The characteristics of this cohort are displayed in (Supplementary Table 11). Comparative analysis was conducted with renal metabolite profiles from mice (4M, 12M, and 24M), to translate these to human urinary findings with renal metabolic changes.

**Fig. 10:**
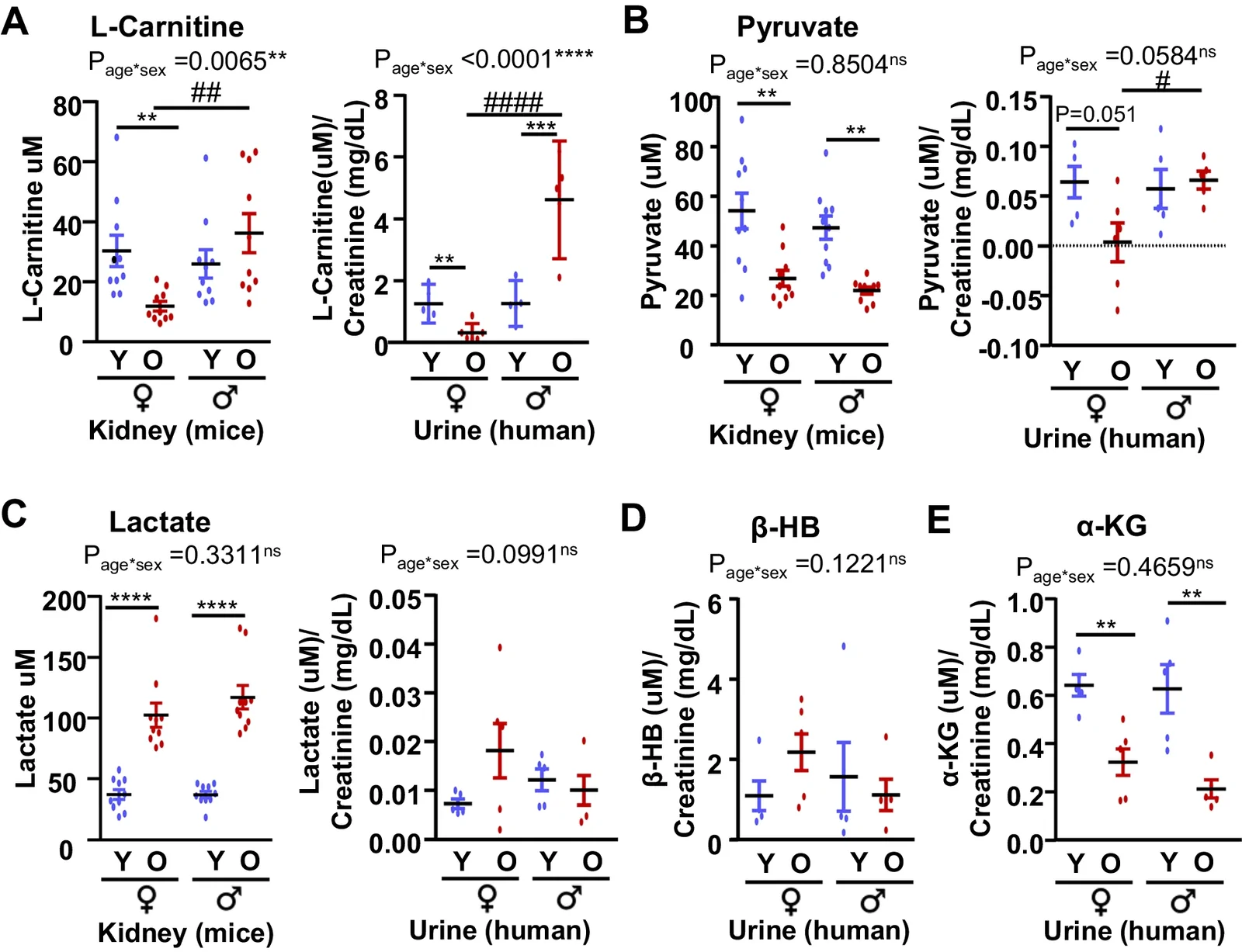
Age- and sex-associated differences in human urinary metabolite levels obtained from healthy individuals. Levels of (**A**) L-carnitine, (**B**) pyruvate, (**C**) lactate, (**D**) α-KG and (**E**) β-HB in mice kidney tissues (n = 10/group) and human urine samples (n = 5/group). Human urinary levels of metabolites were normalized with urine creatinine levels. Two-way ANOVA with Tukey’s multiple comparison test was performed to study the effect of age within same sex (**p < 0.01, ***p < 0.001 ****p < 0.0001) (age differences, same sex) and effect of sex in age-matched groups (#p < 0.05, ##p < 0.01, ####p < 0.0001), and the interaction of age and sex (P_age*sex_) are presented in the graph. 30–45 years, premenopausal females and age-matched men, young (Y) and 60 + years; postmenopausal females and age-matched men, older adult (O); ns, non-significant. β-HB, β-hydroxybutyrate; αKG, α-ketoglutarate

Renal L-carnitine levels were significantly reduced in 24M female mice compared with both 4M females and 24M males. This trend was reflected in human urine samples, where older women (> 65 years) exhibited markedly lower urinary L-carnitine levels compared to both younger females (< 45 years) and older males (> 65 years) (Fig. 10A). These findings suggest a sex-specific decline in mitochondrial fatty acid transport capacity with aging, particularly in females. Renal pyruvate levels decreased significantly in aged mice of both sexes (Fig. 10B). In humans, this reduction was observed only in the urine of older women, indicating that mitochondrial oxidative metabolism may be disproportionately impaired in aging females. While renal lactate levels declined significantly with age in both male and female mice, no corresponding changes were detected in human urinary lactate levels across age or sex groups (Fig. 10C). Levels of β-HB showed no significant age-related differences in human urine (Fig. 10D) while renal β-HB levels declined significantly in aged mice (Fig. 8C). The lack of significant age-related changes in urinary β-HB levels in humans, despite a marked decline in renal β-HB content in aged mice, may suggest that local kidney metabolism is altered with aging, even if systemic or excreted levels remain stable. This may reflect a reduced capacity of renal cells to produce or utilize β-HB, potentially due to impaired mitochondrial function or decreased FAO, while filtration and excretion processes preserve β-HB levels in the urine. Alternatively, β-HB in urine may be influenced by hepatic production or peripheral metabolism, masking kidney-specific deficits. Interestingly, urinary α-KG levels decreased significantly with age in both men and women (Fig. 10E), whereas renal α-KG levels were markedly increased in aged mice of both sexes (Fig. 9E). This plausibly reflects a decrease in renal excretion and a compensatory accumulation of this metabolite in the kidney with age.

Collectively, these findings highlight both conserved and divergent patterns of metabolite regulation with aging between humans and mice. A limitation of this data remains the lack of 24-h urine collections and kidney tissue from humans which may explain some of the differences noted above. The observed sex-specific alterations, particularly in aged women, underscore the importance of considering sex and reproductive status in metabolic and epigenetic studies of aging. The parallel trends in metabolites such as L-carnitine and pyruvate between human urine and mouse kidney suggest potential translational biomarkers of age-associated renal metabolic decline, especially in females.

## Discussion

In this manuscript, we follow upon our previous study, where we have provided evidence of sex-specific differences in age-regulated epigenetic alterations and renal injury markers [19]. In this study we sought to identify the overall metabolic changes during aging in mice and their effect on epigenetic regulations to better understand the mechanisms of age-related kidney pathophysiology. The kidney is a metabolically demanding organ, relying heavily on glucose and mitochondrial FAO to meet its high energy requirements. Our findings reveal a pronounced decline in multiple bioenergetic pathways with aging. Specifically, we observed reduced levels of key FAO-related metabolites, including β-HB and several fatty acylcarnitines (palmitoylcarnitine, myristoylcarnitine, and lauroylcarnitine) as well as TCA cycle intermediates such as citrate and malate. Additionally, pyruvate- an aerobic glycolysis product, and ATP levels were diminished in aged kidneys. These alterations collectively indicate a significant impairment in glycolysis, TCA cycle, and mitochondrial oxidative capacity during renal aging. The impairment of mitochondrial efficiency or biogenesis are well-established features of renal aging and contribute to decreased energy availability and metabolic flexibility. Moreover, reduced FAO and dysregulated lipid uptake contribute to major mechanism leading to kidney injury and diseases [25, 26]. It has been shown that reduction in FAO plays a causative role in the pathogenesis of renal fibrosis during aging [27] and genes involved in FAO were lower in kidney biopsies of CKD patients [28]. Moreover, dysfunctional mitochondria might produce harmful reactive oxygen species therefore, switching to cytosolic ATP production might be a necessity [29]. Indeed, we observed increased lactate levels, an anaerobic glycolysis product, in aged mice and others have also documented enhanced glycolysis rate during aging in lung [30] and heart tissues [31]. This age-related metabolic switch might be partly influenced by change in sex hormones levels since estrogen and testosterone enhance mitochondrial biogenesis [32]. Notably, we observed decreased pyruvate and significantly increased lactate levels in aged mice kidney of both sexes. However, in human urine, decreased pyruvate levels were only observed in aged females whereas no changes were observed in males. Moreover, protein levels of LDHA, a key glycolytic enzyme that catalyzes the conversion of pyruvate to lactate during anaerobic glycolysis, markedly increased with aging. Increased lactate levels, LDHA expression, and impaired energy metabolism have been linked to albuminuria and kidney injury [33, 34].

The kidneys are essential for maintaining systemic homeostasis and regulating amino acid metabolism. Numerous clinical and preclinical studies have reported alterations in amino acid metabolic profiles in plasma, urine, and renal tissues during aging and in various kidney diseases [35, 36]. A recent study with CKD patients aged between 20 and 96 years has identified glycine, serine, and threonine metabolism, phenylalanine and tyrosine metabolism, and arginine and proline metabolism pathways play a pivotal role in the progression of CKD [36]. In accordance, we have also observed similar results as pathway enrichment analysis revealed significantly enriched pathways in aged mice pertaining to amino acid metabolism, including glycine and serine metabolism, phenylalanine and tyrosine metabolism, arginine and proline metabolism, glutamate metabolism, and valine, leucine and isoleucine degradation (branched-chain amino acid, BCAA) (Supplementary Fig. 4 and 5). Our results demonstrate increased levels of BCAAs, including valine, leucine, and isoleucine in aged mice. BCAAs are mainly associated with aging-related pathways and metabolic health [37, 38]. Recent clinical study has shown that higher levels of BCAA are associated with CKD in type 2 diabetes [38]. We also observed elevated levels of several amino acid-related metabolites in aged mouse kidneys, particularly argininosuccinate, aspartate, and glutamine. Argininosuccinate, a key intermediate in the urea cycle responsible for nitrogen waste removal, showed marked accumulation—suggesting potential impairment in urea cycle function with aging [39].Glutamine has a central role in kidney metabolism by mediating nitrogen clearance, acid–base metabolism, and gluconeogenesis [40, 41]. Accumulation of these compounds with aging points to potential dysregulation in nitrogen handling, including urea cycle and ammonia detoxification pathways which may contribute to a pro-inflammatory or toxic metabolic environment in aged tissues. Moreover, these changes suggest an increased reliance on amino acid catabolism in aged kidneys, which may reflect elevated protein turnover, cellular stress responses, or compensatory energy production mechanisms as mitochondrial efficiency declines with age.

Furthermore, our data shows elevated levels of urea and 8-oxoG, a well-established biomarker of oxidative DNA damage, which highlights the presence of increased oxidative stress in the aging kidney. 8-oxoguanine is used to assess the overall effects of oxidative stress in various diseases [42]. Moreover, 8-oxoG has epigenetic and epitranscriptional roles, which underscores the significance of oxidative modification in redox-mediated control of gene expression [43]. Also, high urea levels have been suggested to cause oxidative stress and 8-oxoG formation in renal cells, which can lead to protein carbonylation and DNA damage, further contributing to renal dysfunction [44].

Considering that sex differences influence aging physiology and pathophysiology, which is reflective of changes in metabolic pathways, there is relatively limited information in this regard. Our results from multivariate analyses (PLS-DA biplot) and univariate analysis between sexes imply that both chronological aging and sex exert dominant, non-overlapping effects on renal metabolic profiles. Although, young mice exhibited fewer sex-defined changes in metabolite levels, these differences progressively increased with age in 12M female and male mice groups. Twelve-month-old females showed significantly upregulated metabolites, including bile acids, purine/pyrimidine derivatives, and fatty acid metabolites, while fatty acylcarnitines were downregulated relative to 12M males. This shift suggests a more substantial divergence in energy metabolism and renal handling of lipids and nucleotides. The upregulation of bile acids could indicate sex-based differences in lipid digestion and detoxification pathways, while reduced acylcarnitines in females may reflect differences in mitochondrial FAO or carnitine-dependent transport. Notably, several sex-specific markers emerged with aging, as aged females exhibited elevated levels of amino acids and significantly reduced metabolites, like lysophospholipids, as well as creatinine and carnitine, compared to aged males. We also observed lower urinary carnitine in aged women compared with young women and aged men and increased levels in older men compared with young men. Carnitine is an essential metabolite for transporting long-chain fatty acids into mitochondria and for promoting β-oxidation to provide energy for cellular function and prevents mitochondrial dysfunction [45]. Moreover, carnitine deficiency promotes fibrosis of renal tissue [46]. Besides sex-specific changes in carnitine levels with aging, we also observed sex differences in the enzyme, CPT1A protein levels as it was significantly lower in aged females compared with young females and aged male mice. CPT1A is a rate-limiting enzyme in the FAO pathway which catalyzes the synthesis of acylcarnitine from long chain fatty acyl CoA and carnitine for the process of β-oxidation and ATP generation. Overexpression of CPT1a in kidney tubules significantly reduced renal fibrosis and protect against kidney function deterioration by restoring FAO function, thereby preventing mitochondrial dysfunction [47]. Conversely, aged males showed increased levels of inflammatory oxylipins and lipid signaling molecules. Oxylipins are key lipid mediators that play significant roles in kidney function and disease, contributing to inflammation, oxidative stress, and the progression of renal disease [48, 49]. An earlier study in rats fed with higher linoleic acid diet exhibited sex differences in kidney, liver, and serum levels of oxylipins, where 40% of oxylipins detected were higher in males [50]. Furthermore, our results show elevated urea cycle metabolites in males which implies enhanced nitrogen disposal and amino acid turnover compared with aged females. These findings suggest that changes in sex hormone levels with aging defines more metabolic pathways. Estrogen levels in females and testosterone in males peak around the age of 6 months in mice and decline subsequently during aging [51, 52]. The elevated amino acid levels in aged females may suggest enhanced protein catabolism or altered amino acid handling, potentially linked to sex-specific aging trajectories or compensatory renal mechanisms. These differential metabolic signatures provide further evidence for sex-specific vulnerabilities in renal aging—namely, bioenergetic decline in females versus inflammatory remodeling in males.

Emerging evidence shows that epigenetic enzymes facilitating chemical modifications of the chromatin are metabolically regulated and several metabolites act as cofactors for these enzymes as reviewed [53, 54]. From a physiological perspective, the epigenetic response to metabolism plays a critical role in nutrient sensing and reprogramming. However, the evidence that changes in metabolites in response to various physiological stimuli thereby influences enzymes implicated in chromatin remodeling is limited. Our recent study has demonstrated that age and biological sex differentially regulates epigenetic enzymes and histone modifications [19]. Our present data provides evidence that some of the epigenetic enzyme activities such as acetylation/deacetylation and methylation/demethylation were modulated by the levels of their cofactors. Our metabolomics data revealed significant higher levels of ac-CoA in aged mice of both sexes; however, total acetylated histones were markedly reduced in aged mice than their younger counter parts. Acetyl-CoA localizes in the cytoplasm and nucleus of mammalian cells, is generated from oxidation of glucose and FAs, enters the TCA cycle, and contributes to cellular energy supply [55]. Acetyl-CoA metabolism and histone acetylation are tightly linked in the regulation of aging and lifespan [56]. In accordance with our result, earlier study has shown increased ac-CoA levels by roughly 60% from 3 to 30 M of age in mouse liver [57] and contrarywise, a decrease in global acetylation has been observed in senescent cells and aging [58, 59]. We also observed significantly lower levels of β-HB, an anti-aging metabolite and an endogenous HDAC inhibitor, and increased class I HDACs protein levels with aging. In accordance with our findings, earlier studies have demonstrated that β-HB naturally inhibits HDACs in a dose-dependent manner, including HDAC1, 2, and 3 and alleviate oxidative stress [60]. Moreover, β-HB has been suggested to protect against energy depletion, oxidative stress, inflammation, autophagy, and apoptosis by inhibition of HDACs [61].

Additionally, we observed changes in metabolite cofactor levels and their respective enzymes involved in DNA and histone demethylation. Our data showed higher levels of α-KG and increased activities of its dependent enzymes, including the jumonji domain–containing HDMs and Tet proteins responsible for active DNA demethylation in aged mice of both sexes. Aging is generally marked by global DNA hypomethylation, but selective CpG islands can be hypermethylated [62]. However, our results from human urine analysis show significantly decreased levels of α-KG in older adults of both sexes. Accordingly, reduced circulatory concentration of α-KG was observed in aging mice [63] and in aging humans [64]. Alpha-ketoglutarate is a key intermediate of various metabolic pathways including TCA cycle, amino acids metabolism, and collagen biosynthesis and its dysregulation has been associated with declined renal function [64, 65]. Interestingly, α-KG has been shown to delay onset of aging phenotypes in mice [66, 67]. Flavin adenine dinucleotide plays a crucial role in the citric acid cycle and electron transport chain, which are central to cellular energy production. Our metabolomics data revealed a significant increase in FAD levels and an increase in enzymatic activity of LSD1 with aging. Lysine-specific histone demethylase 1 A (LSD1) is a FAD-dependent demethylase that erases the mono-, and dimethylation of H3K4, resulting in the suppression of target gene transcription [68]. Studies indicate that LSD1 is upregulated in renal injury and fibrotic conditions, and that its inhibition may help mitigate these pathologies [69, 70].

Interestingly, our results demonstrate accumulation of epigenetically modified amino acid metabolites with aging, including N-acetylated and methylated amino acids such as N-methylglutamate, N-methyglycine, N, N-dimethylarginine, 3-methylhistidine, N-acetylglutamine, N-acetylglutamate, methylglycine, N-acetylserine, and others. It has been proposed that these modified compounds, defined as ‘epimetabolites’ have functions distinct from classical functions in anabolism or catabolism [71]. Epimetabolites are structurally like their canonical metabolic counterparts but differ by reversible modifications such as methylation or acetylation. Methylation of well-known metabolites like glycine, arginine, or nicotinamide gives rise to epimetabolites that play important roles in cellular signaling and physiological regulation. For example, N-methylglycine, an oncometabolite stimulates invasion and aggressive phenotype in prostate cancer cells [72], was initially discovered through untargeted metabolomics. Asymmetric and symmetric dimethyl arginine (ADMA and SDMA) are produced by the repeated methylation of arginine residues by protein arginine methyltransferases. A meta-analysis involving more than 4,000 subjects has revealed that ADMA levels are significantly associated with an increased risk of coronary artery disease [73]. Urinary SDMA levels, quantified by targeted LC–MS/MS, has been suggested as a potential biomarker for renal insufficiency [74]. N-acetylglutamate is a precursor of arginine and an activator of the urea cycle an essential cofactor of carbamoyl phosphate synthetase 1 in the urea cycle [75]. In our study, accumulation of modified amino acids indicates increased post-translational modifications and potential epigenetic regulation which are associated with pathophysiology of kidney and other organs.

In conclusion, our findings from renal metabolomic analyses of aged female and male mice kidney tissues collectively reflect a metabolic remodeling in the aging kidney. It is characterized by reduced lipid utilization and increased amino acid degradation, a progressive impairment of mitochondrial FAO and central energy metabolism, and dysregulated nitrogen recycling which may serve as both a marker and a mechanistic contributor to renal aging. Our results also highlight an aging-associated increase in epimetabolites, which needs to be studied further, as these may prove to be important as specific biomarkers for disease states. To our knowledge, this is the first report which provides compelling evidence of association between metabolic alterations and epigenetic enzymatic activities as metabolites are critical cofactors of these enzymes, which in turn regulate gene expression. Additionally, age-specific changes in metabolic demand may underlie age-related vulnerability to renal dysfunction and could inform the development of sex- and age-specific biomarkers or therapeutic targets for preserving kidney health across the lifespan.

## Limitations

This work helps to establish the fact that renal metabolite cofactors can modulate epigenetic enzyme activity which in turn would regulate gene expression in sex- and age-specific manner. There are some limitations in this study. First, the metabolomics study and the renal epigenetic markers were quantified in the whole kidney homogenate to draw mechanistic insights, however, it does not reflect cell-specific information as structure and function of the kidneys are highly compartmentalized and kidneys have high metabolic rate. Future studies are planned to further investigate the cell type-specific changes in renal metabolic pathways and epigenome with aging. Also, we were unable to correlate kidney metabolite data with urine because the kidney tissues were sourced from the NIA, and urine samples are not available in the NIA biobank. Second, to explore the translational context of some of the renal metabolites, we evaluated human urine samples. The numbers were relatively small but did corroborate our findings. However, these samples were not 24-h urine samples which are considerably difficult to obtain from humans without any clinical indications. In addition, while we evaluate kidney metabolite in mice, we did not have access to human kidney tissue. While our study plausibly identifies a few urine biomarkers for an aging kidney, larger biomarker trials are required to validate these results.

## Supplementary Information

Below is the link to the electronic supplementary material.

ESM 1 (DOCX 2.29 MB)

## Data Availability

The datasets generated during and/or analyzed during the current study are available from the corresponding author on reasonable request.
