# Supplementary material for: Aging kidney is associated with metabolic rewiring and epigenetic reprogramming
Source: GeroScience. 2026 Jan 7;48(6):7769–94. doi: 10.1007/s11357-025-02071-0 (PMC13638952; doi:10.1007/s11357-025-02071-0)
Supplement: Supplementary file 1 [file 11357_2025_2071_MOESM1_ESM.docx]

**Aging Kidney is Associated with Metabolic Rewiring and Epigenetic Reprogramming**

Pragney Deme^1^, Rhea Bhargava^2^, Heddwen L Brooks^1^, Norman J Haughey^1^, and Prerna Kumar^1^

^1^Department of Physiology, School of Medicine, Tulane University, New Orleans, Louisiana, USA

^2^Department of Medicine Nephrology and Hypertension, School of Medicine, Tulane University, New Orleans, Louisiana, USA

Running Head: Age-specific alterations in renal metabolome

Address for Correspondence:

Prerna Kumar, Ph.D.

Department of Physiology, 8639

1430 Tulane Ave, New Orleans, LA 70112, USA

Tel: (504) 988-2601

Fax: (504) 988-2675

Email: [pkumar@tulane.edu](mailto:pkumar@tulane.edu)

ORCID: <https://orcid.org/0000-0002-5989-1878>

**Supplementary Table 1**. The list of significantly altered female renal metabolites identified between 12- and 4-month age groups in mice.

| Metabolite Level 12M vs 4M | FC | p_value | q_value |
| --- | --- | --- | --- |
| Lyso PE 20:1 | 1.7171 | 0.0089672 | 1 |
| Cysteine | 0.65965 | 0.029531 | 1 |
| Pimelylcarnitine | 0.61261 | 0.02021 | 1 |
| Decanoylcarnitine | 0.4906 | 0.046993 | 1 |
| Lauroylcarnitine | 0.45326 | 0.0040885 | 1 |

**Supplementary Table 2.** The list of significantly altered female renal metabolites identified between 24- and 4-month age groups in mice.

| Metabolite Level 24M vs 4M | FC | p_value | q_value |
| --- | --- | --- | --- |
| N-methylarginine | 5.062 | 4.50E-09 | 0.000192 |
| Uracil | 4.5825 | 5.29E-09 | 0.000192 |
| N-Methyglutamate | 4.4837 | 3.93E-09 | 0.000192 |
| Indole 3-acetate | 3.9902 | 2.73E-08 | 0.000192 |
| Guanidinoacetate | 3.8534 | 6.50E-05 | 0.003001 |
| Taurochenodeoxycholate | 3.6566 | 5.45E-06 | 0.000192 |
| 8-oxoguanine | 3.6083 | 4.48E-08 | 0.000192 |
| Glycochenodeoxycholate | 3.5734 | 9.11E-07 | 0.000192 |
| Glycerophosphocholine | 3.2393 | 5.17E-05 | 0.001667 |
| Glutamine | 2.9011 | 6.68E-05 | 0.002376 |
| Gamma-Aminobutyrate | 2.8561 | 6.10E-09 | 0.000192 |
| Urate | 2.8532 | 0.001119 | 0.011212 |
| Sebacate | 2.7852 | 1.79E-08 | 0.000192 |
| Lactate | 2.7681 | 1.05E-06 | 0.000192 |
| p-cresol glucuronide | 2.7658 | 3.16E-05 | 0.001667 |
| 3-methylhistidine | 2.6175 | 3.12E-05 | 0.000667 |
| Lyso PC 20:5 | 2.4813 | 0.028503 | 0.146947 |
| N-Acetylphenylalanine | 2.4146 | 0.00037928 | 0.005984 |
| Methylglycine | 2.4002 | 0.00053343 | 0.000667 |
| Palmitate | 2.2797 | 1.11E-05 | 0.000192 |
| leucine | 2.2669 | 0.00024448 | 0.003001 |
| Phenylalanine | 2.2439 | 9.60E-05 | 0.004168 |
| Cotinine N-oxide | 2.1991 | 4.85E-05 | 0.002376 |
| Stearate | 2.1818 | 0.0001082 | 0.003001 |
| Indole 3-lactate | 2.0412 | 5.60E-05 | 0.001667 |
| Lyso PE 20:1 | 2.0058 | 0.029028 | 0.141241 |
| Deoxyguanosine | 1.9223 | 0.00087404 | 0.004168 |
| 2-methylhippurate | 1.9182 | 0.0037498 | 0.019563 |
| lsoleucine | 1.8542 | 0.00016473 | 0.003001 |
| Aspartate | 1.8526 | 0.032659 | 0.170569 |
| PGE2 | 1.8099 | 0.0019736 | 0.03244 |
| S-MethylCysteine | 1.8083 | 0.0012876 | 0.011212 |
| Creatinine | 1.795 | 0.0038322 | 0.03244 |
| Glutathione | 1.7414 | 0.042196 | 0.146947 |
| Asparagine | 1.7008 | 0.03051 | 0.146947 |
| Hippurate | 1.6675 | 0.031685 | 0.146947 |
| S-Sulfocysteine | 1.6484 | 0.013466 | 0.040522 |
| Taurine | 1.6476 | 0.039245 | 0.141241 |
| N-glycylproline | 1.64 | 0.0043246 | 0.019563 |
| Tyrosine | 1.6337 | 0.0035965 | 0.025649 |
| Myristate | 1.6192 | 0.0056483 | 0.057731 |
| Lyso PC 18:1 | 1.6063 | 0.005189 | 0.057731 |
| Uridine | 1.6028 | 0.010556 | 0.103198 |
| alpha-Ketoglutarate | 1.5716 | 0.0034739 | 0.019563 |
| Argininosuccinate | 1.5711 | 0.0034837 | 0.040522 |
| N-Carbamoyl-alanine | 1.5585 | 0.01684 | 0.072198 |
| Valine | 1.5289 | 0.0092831 | 0.057731 |
| N,N-Dimethyarginine | 1.5196 | 0.022197 | 0.087419 |
| LTB4 | 1.4633 | 0.028599 | 0.170569 |
| N-Acetylserine | 1.4415 | 0.015865 | 0.222874 |
| Lyso PC 18:0 | 1.3596 | 0.029152 | 0.146947 |
| Glutarylcarnitine | 1.3035 | 0.033021 | 0.120983 |
| Azelate | 1.2133 | 0.042326 | 0.146947 |
| Cysteine | 0.68433 | 0.046045 | 0.282364 |
| Malate | 0.68426 | 0.014002 | 0.050317 |
| Purine | 0.65196 | 0.02705 | 0.120983 |
| Glutarate | 0.65141 | 0.0279 | 0.146947 |
| Lyso PA 18:0 | 0.61263 | 0.023888 | 0.103198 |
| Adenosine triphosphate | 0.6039 | 0.040854 | 0.103198 |
| Lauroylcarnitine | 0.56283 | 0.022673 | 0.141241 |
| Pyruvate | 0.49736 | 0.0024772 | 0.050317 |
| Oleate | 0.47456 | 0.0015279 | 0.011212 |
| Carnitine | 0.43335 | 0.0082466 | 0.057731 |
| Guanosine | 0.40217 | 0.0045002 | 0.057731 |
| Myristoylcarnitine | 0.32125 | 0.00038368 | 0.003001 |
| Beta-hydroxybutyrate | 0.31438 | 5.76E-07 | 0.000192 |
| Palmitoylcarnitine | 0.1613 | 3.37E-08 | 0.000192 |

**Supplementary Table 3.** The list of significantly altered female renal metabolites identified between 24- and 12-month age groups in mice.

| Metabolite Level 24M vs 12M | FC | p_value | q_value |
| --- | --- | --- | --- |
| Uracil | 5.425 | 1.12E-09 | 0.000754 |
| Urate | 4.315 | 2.32E-05 | 0.000754 |
| N-methylarginine | 4.2539 | 1.57E-07 | 0.000754 |
| 8-oxoguanine | 4.2243 | 1.40E-07 | 0.000754 |
| Indole 3-acetate | 4.0133 | 7.51E-08 | 0.000754 |
| Glutamine | 3.5914 | 8.45E-07 | 0.000754 |
| N-Methyglutamate | 3.5122 | 7.42E-08 | 0.000754 |
| Glycerophosphocholine | 3.4307 | 5.96E-05 | 0.002011 |
| Gamma-Aminobutyrate | 3.3311 | 6.94E-07 | 0.000754 |
| Deoxyguanosine | 3.119 | 4.48E-06 | 0.001242 |
| Glycochenodeoxycholate | 3.0919 | 1.11E-05 | 0.001242 |
| Cotinine N-oxide | 3.0066 | 1.21E-06 | 0.001242 |
| Lactate | 2.7535 | 1.59E-07 | 0.000754 |
| Sebacate | 2.7136 | 7.66E-09 | 0.000754 |
| p-cresol glucuronide | 2.5933 | 0.00021812 | 0.00743 |
| Taurochenodeoxycholate | 2.5746 | 0.00064918 | 0.003079 |
| Decanoylcarnitine | 2.456 | 0.0037134 | 0.026673 |
| leucine | 2.3866 | 0.00017871 | 0.002011 |
| PGE2 | 2.3442 | 0.00011847 | 0.000754 |
| Guanidinoacetate | 2.2476 | 0.005998 | 0.098762 |
| lsoleucine | 2.1225 | 0.00017493 | 0.004873 |
| S-Sulfocysteine | 2.0951 | 0.0021052 | 0.016383 |
| N-Acetylphenylalanine | 2.0704 | 0.0052316 | 0.020211 |
| Creatinine | 2.0663 | 0.00061604 | 0.020211 |
| MG 16:1 | 2.0372 | 0.045591 | 0.196809 |
| Palmitate | 2.0148 | 1.40E-06 | 0.000754 |
| Indoleacetylglutamine | 1.986 | 0.024699 | 0.118372 |
| Glutathione | 1.9736 | 0.025187 | 0.196809 |
| Methylglycine | 1.9574 | 0.00024758 | 0.003079 |
| Myristate | 1.9196 | 0.0013776 | 0.035022 |
| 3-methylhistidine | 1.9149 | 0.00053612 | 0.004873 |
| S-MethylCysteine | 1.9143 | 0.0042715 | 0.026673 |
| Stearate | 1.8429 | 0.0018921 | 0.020211 |
| Uridine | 1.8058 | 0.002659 | 0.020211 |
| Valine | 1.7947 | 0.0017288 | 0.020211 |
| Phenylalanine | 1.7937 | 0.0061305 | 0.076378 |
| Tyrosine | 1.7848 | 3.95E-05 | 0.002011 |
| Asparagine | 1.7712 | 0.030181 | 0.141403 |
| Argininosuccinate | 1.7573 | 0.00021134 | 0.003079 |
| N-Acetylserine | 1.6997 | 0.0040983 | 0.058528 |
| Choline | 1.6946 | 0.00044048 | 0.020211 |
| N-glycylproline | 1.6688 | 0.0055645 | 0.045915 |
| Indole 3-lactate | 1.6495 | 0.0044797 | 0.035022 |
| 2-methylhippurate | 1.6405 | 0.028687 | 0.118372 |
| Betaine | 1.5939 | 0.04749 | 0.175968 |
| N-Carbamoyl-alanine | 1.5641 | 0.029924 | 0.118372 |
| Docosahexaenoate | 1.5535 | 0.047652 | 0.274359 |
| Azelate | 1.5488 | 0.0010606 | 0.026673 |
| N,N-Dimethyarginine | 1.4603 | 0.036886 | 0.196809 |
| Glutarylcarnitine | 1.4142 | 0.015351 | 0.118372 |
| Lyso PC 18:1 | 1.4019 | 0.020276 | 0.196809 |
| Pyruvate | 0.63908 | 0.0066049 | 0.058528 |
| Lyso PE 18:1 | 0.60678 | 0.046814 | 0.196809 |
| Oleate | 0.60425 | 0.0067074 | 0.058528 |
| Uridine triphosphate | 0.55963 | 0.038041 | 0.196809 |
| MG 18:1 | 0.55139 | 0.023442 | 0.141403 |
| Adenosine triphosphate | 0.54115 | 0.0031833 | 0.011312 |
| Succinate | 0.4586 | 0.0059074 | 0.035022 |
| Carnitine | 0.44702 | 0.04684 | 0.236597 |
| Guanosine | 0.42363 | 0.0050822 | 0.045915 |
| Myristoylcarnitine | 0.38591 | 0.00018181 | 0.002011 |
| Beta-hydroxybutyrate | 0.30037 | 1.14E-06 | 0.000754 |
| Palmitoylcarnitine | 0.13927 | 1.29E-08 | 0.000754 |

**Supplementary Figure 1**

**Supplementary Figure1. Pathway enrichment analysis of age-associated metabolites in female kidneys (24 vs 4 months).** This figure illustrates the metabolic pathways significantly enriched among metabolites altered between 24- and 4-month-old female mice. Pathway enrichment was performed using over-representation analysis based on the list of significantly changed metabolites (p < 0.05) identified from pairwise age comparisons. Each bar represents a metabolic pathway, with the enrichment ratio shown on the x-axis, reflecting the proportion of altered metabolites relative to the total number of metabolites annotated within that pathway. Higher enrichment ratios indicate stronger over-representation. Bars are color-coded according to the corresponding p-values, with deeper red tones representing pathways with higher statistical significance, and lighter yellow/orange colors indicating less significant enrichment.

**Supplementary Figure 2**

**Female 4M**

**Female 12M**

**Female 24M**

**Supplementary Figure 2.** Heatmap illustrating the metabolites that were significantly altered during renal aging in female mice and their relative abundances across 4-, 12-, and 24-month age groups. The color gradient reflects relative abundance, with red indicating higher levels and blue indicating lower levels. n=10 (4M); n=8 (12M); n=10 (24M)

**Supplementary Table 4.** The list of significantly altered male renal metabolites identified between 12- and 4-month age groups in mice.

| Metabolite Level 12M vs 4M | FC | p_value | q_value |
| --- | --- | --- | --- |
| Lyso PE 20:5 | 2.0851 | 0.0023908 | 0.141478 |
| Docosanoylcarnitine | 2.0016 | 0.023665 | 0.49491 |
| Glycerophosphocholine | 1.9519 | 0.0010505 | 0.141478 |
| 2-Hydroxyhexanoate | 1.9161 | 0.00029146 | 0.141478 |
| Lyso PC 18:1 | 1.8258 | 0.024613 | 0.39886 |
| Lyso PA 18:1 | 1.7842 | 0.023939 | 0.49491 |
| Octanoylcarnitine | 1.7729 | 0.027856 | 0.49491 |
| Urea | 1.6365 | 0.037144 | 0.686896 |
| Palmitate | 1.6315 | 0.020663 | 0.39886 |
| 3-hydroxyisovalerate | 1.5829 | 0.019955 | 0.49491 |
| Suberate | 0.74428 | 0.019469 | 0.557692 |
| 7-methylxanthine | 0.73461 | 0.028252 | 0.527903 |
| N-Acetylaspartylglutamate | 0.72996 | 0.036482 | 0.557692 |
| Lyso PC 20:4 | 0.70403 | 0.037539 | 0.557692 |
| 2-oxoadipate | 0.65596 | 0.0024966 | 0.141478 |
| Methionine | 0.65305 | 0.046822 | 0.527903 |
| 5-methylcytosine | 0.6177 | 0.028364 | 0.49491 |
| Purine | 0.60397 | 0.00064333 | 0.141478 |
| 5-HETE | 0.60367 | 0.024027 | 0.557692 |
| Carnitine | 0.60149 | 0.045661 | 0.527903 |
| 3-Formylindole | 0.59134 | 0.012301 | 0.384615 |
| Lyso PA 18:0 | 0.54424 | 0.038509 | 0.686896 |
| Lyso PC 18:2 | 0.50595 | 0.0034975 | 0.239316 |
| Lyso PE 18:3 | 0.46605 | 0.015381 | 0.474057 |
| N-Acetylalanine | 0.41424 | 0.017251 | 0.49491 |

**Supplementary Table 5.** The list of significantly altered male renal metabolites identified between 24- and 4-month age groups in mice.

| Metabolite Level 24M vs 4M | FC | p_value | q_value |
| --- | --- | --- | --- |
| N-Methyglutamate | 6.1375 | 9.27E-12 | q_value |
| Urate | 6.0549 | 4.02E-09 | 0.000139 |
| Uracil | 5.6528 | 2.33E-10 | 0.000139 |
| Taurochenodeoxycholate | 5.1323 | 3.85E-08 | 0.000139 |
| Glycerophosphocholine | 3.9313 | 9.27E-07 | 0.000139 |
| Phenylalanine | 3.8805 | 6.56E-08 | 0.000139 |
| Gamma-Aminobutyrate | 3.6831 | 2.65E-08 | 0.000139 |
| N-Acetylphenylalanine | 3.3956 | 3.54E-05 | 0.000139 |
| Homocysteine | 3.3591 | 0.0016848 | 0.00037 |
| 3-methylhistidine | 3.3462 | 2.51E-06 | 0.018702 |
| 8-oxoguanine | 3.223 | 1.65E-07 | 0.000208 |
| Lyso PE 18:2 | 3.2091 | 0.018367 | 0.000139 |
| Sebacate | 3.1777 | 8.52E-12 | 0.080094 |
| Lactate | 3.164 | 6.98E-09 | 0.000139 |
| Palmitate | 3.0925 | 2.79E-07 | 0.000139 |
| Glycochenodeoxycholate | 3.0739 | 1.68E-08 | 0.000139 |
| N-Acetylglutamate | 3.0736 | 2.29E-09 | 0.000139 |
| Creatinine | 2.8852 | 1.40E-05 | 0.000139 |
| Guanidinoacetate | 2.8314 | 0.00034403 | 0.000208 |
| Methylglycine | 2.6707 | 2.86E-06 | 0.00869 |
| Glutamine | 2.5264 | 2.35E-05 | 0.000208 |
| Stearate | 2.4795 | 1.12E-05 | 0.001 |
| PGE2 | 2.4256 | 9.54E-05 | 0.000139 |
| Glycocholate | 2.4062 | 0.021257 | 0.000139 |
| N-Acetylserine | 2.3461 | 9.38E-05 | 0.080094 |
| N-methylarginine | 2.3303 | 8.00E-07 | 0.003126 |
| Lyso PE 20:5 | 2.2883 | 0.0023083 | 0.000139 |
| Indole 3-acetate | 2.1721 | 6.91E-05 | 0.018702 |
| lsoleucine | 2.1484 | 1.49E-05 | 0.002273 |
| Valine | 2.1173 | 4.04E-05 | 0.00037 |
| 2-methylhippurate | 2.0794 | 0.00081307 | 0.001 |
| S-MethylCysteine | 2.0767 | 8.79E-05 | 0.003126 |
| Tyrosine | 2.0574 | 0.00023953 | 0.001 |
| Lyso PC 18:1 | 2.052 | 0.00053547 | 0.003126 |
| Argininosuccinate | 2.048 | 5.17E-06 | 0.011491 |
| p-cresol glucuronide | 2.0406 | 0.0017163 | 0.000208 |
| LTB4 | 1.9618 | 0.0016349 | 0.006383 |
| alpha-Ketoglutarate | 1.9335 | 0.0011168 | 0.00869 |
| S-Sulfocysteine | 1.9127 | 0.0022949 | 0.004528 |
| Lyso PE 18:1 | 1.9044 | 0.016087 | 0.018702 |
| N-Acetylleucine | 1.8344 | 0.011301 | 0.071392 |
| Azelate | 1.7927 | 0.0262 | 0.080094 |
| 9,10-DiHODE | 1.7699 | 0.048439 | 0.018702 |
| TXB2 | 1.7461 | 0.0047127 | 0.131479 |
| 2-Hydroxyhexanoate | 1.7383 | 0.033755 | 0.060595 |
| Cotinine N-oxide | 1.7376 | 0.0037866 | 0.131479 |
| Deoxyguanosine | 1.7343 | 0.023191 | 0.030392 |
| N-Methylaspartate | 1.705 | 0.025272 | 0.142475 |
| Myoinositol | 1.6898 | 0.0011373 | 0.095058 |
| Betaine | 1.6889 | 0.018045 | 0.011491 |
| Cysteinylglycine | 1.6695 | 0.0031219 | 0.071392 |
| Myristate | 1.666 | 0.0001651 | 0.030392 |
| Docosanoylcarnitine | 1.6453 | 0.016592 | 0.002273 |
| N-Myristoyglycine | 1.6168 | 0.037987 | 0.080094 |
| N-glycylproline | 1.5892 | 0.047439 | 0.165409 |
| Uridine | 1.5805 | 0.00088382 | 0.219228 |
| N-stearoyltaurine | 1.5299 | 0.032278 | 0.015469 |
| N-Acetylglutamine | 1.5268 | 0.019432 | 0.131479 |
| N,N-Dimethyarginine | 1.5257 | 0.01479 | 0.071392 |
| Arachidonate | 1.4943 | 0.032387 | 0.038204 |
| Urea | 1.4924 | 0.019431 | 0.11538 |
| 3-hydroxyisovalerate | 1.4739 | 0.018644 | 0.165409 |
| MG 16:0 | 1.4595 | 0.022856 | 0.095058 |
| leucylglycine | 1.447 | 0.029569 | 0.080094 |
| Lyso PI 16:0 | 1.412 | 0.0054754 | 0.142475 |
| N-Arachidonoylglycine | 1.4037 | 0.042518 | 0.018702 |
| Choline | 1.3196 | 0.01048 | 0.131479 |
| Suberate | 1.2551 | 0.0035293 | 0.030392 |
| Oleate | 0.73647 | 0.017697 | 0.024496 |
| Lysine | 0.69561 | 0.039217 | 0.095058 |
| Purine | 0.65907 | 0.011511 | 0.219228 |
| Methionine | 0.58498 | 0.011213 | 0.071392 |
| PGE3 | 0.56585 | 0.017434 | 0.038204 |
| N-Acetylalanine | 0.53613 | 0.041304 | 0.080094 |
| Lauroylcarnitine | 0.53217 | 0.003651 | 0.131479 |
| Pimelylcarnitine | 0.49349 | 0.030567 | 0.001533 |
| Pyruvate | 0.46342 | 8.91E-06 | 0.142475 |
| Malate | 0.44795 | 4.50E-05 | 0.000208 |
| Myristoylcarnitine | 0.35226 | 3.92E-06 | 0.000208 |
| Beta-hydroxybutyrate | 0.27839 | 2.59E-09 | 0.00037 |
| Palmitoylcarnitine | 0.1687 | 7.08E-08 | 0.000139 |

**Supplementary Table 6.** The list of significantly altered male renal metabolites identified between 24- and 12-month age groups in mice.

| Metabolite Level 24M vs 12M | FC | p_value | q_value |
| --- | --- | --- | --- |
| Urate | 6.2457 | 7.27E-08 | 0.000528 |
| Taurochenodeoxycholate | 5.5062 | 3.84E-07 | 0.000528 |
| N-Methyglutamate | 4.725 | 4.78E-10 | 0.000528 |
| Uracil | 4.6949 | 8.02E-07 | 0.000918 |
| Gamma-Aminobutyrate | 3.909 | 4.26E-08 | 0.000528 |
| N-Acetylphenylalanine | 3.9039 | 5.36E-06 | 0.000528 |
| N-Acetylglutamate | 3.8163 | 5.24E-08 | 0.000528 |
| Lyso PE 18:3 | 3.6854 | 0.00097541 | 0.012182 |
| Lactate | 3.3174 | 7.13E-08 | 0.000528 |
| Creatinine | 3.2892 | 1.09E-05 | 0.001624 |
| 8-oxoguanine | 3.1736 | 2.25E-06 | 0.000528 |
| Indole 3-acetate | 3.1213 | 2.26E-07 | 0.000528 |
| Sebacate | 3.1088 | 1.26E-07 | 0.000528 |
| Phenylalanine | 3.0748 | 7.69E-06 | 0.000918 |
| Lyso PE 18:2 | 2.9978 | 0.039407 | 0.163582 |
| 3-methylhistidine | 2.9369 | 0.0001482 | 0.00264 |
| Methylglycine | 2.905 | 1.98E-06 | 0.000528 |
| Glycochenodeoxycholate | 2.7919 | 1.43E-07 | 0.000528 |
| S-Sulfocysteine | 2.592 | 0.000465 | 0.00905 |
| Glutamine | 2.4888 | 4.39E-05 | 0.00264 |
| Stearate | 2.4765 | 1.55E-08 | 0.000528 |
| PGE2 | 2.4704 | 8.29E-06 | 0.000528 |
| Lyso PA 18:0 | 2.4391 | 0.0010622 | 0.00905 |
| Argininosuccinate | 2.4025 | 8.19E-08 | 0.000528 |
| Tyrosine | 2.3997 | 0.00018891 | 0.00905 |
| Carnitine | 2.3183 | 0.0076929 | 0.050798 |
| N-methylarginine | 2.3048 | 6.95E-06 | 0.000528 |
| Deoxyguanosine | 2.24 | 0.0028813 | 0.022034 |
| Hippurate | 2.18 | 0.0086108 | 0.111529 |
| 2-methylhippurate | 2.101 | 0.002091 | 0.012182 |
| N-Acetylserine | 2.0604 | 0.00055654 | 0.004369 |
| Glycerophosphocholine | 2.0141 | 0.005643 | 0.022034 |
| Guanidinoacetate | 1.9876 | 0.017871 | 0.111529 |
| Lyso PC 18:2 | 1.9552 | 0.029539 | 0.076461 |
| Palmitate | 1.8955 | 0.00028642 | 0.001624 |
| Cotinine N-oxide | 1.8926 | 0.0033315 | 0.050798 |
| S-MethylCysteine | 1.886 | 0.0014799 | 0.006687 |
| Valine | 1.8834 | 0.0021404 | 0.016843 |
| N-glycylproline | 1.8513 | 0.011504 | 0.050798 |
| N-Acetylleucine | 1.847 | 0.0058991 | 0.022034 |
| TXB2 | 1.8182 | 0.0053883 | 0.050798 |
| Myoinositol | 1.7939 | 0.00082691 | 0.016843 |
| 2-oxoadipate | 1.7824 | 0.0051577 | 0.016843 |
| p-cresol glucuronide | 1.7469 | 0.019178 | 0.228632 |
| Myristate | 1.7289 | 0.0012257 | 0.00905 |
| N-Acetylaspartylglutamate | 1.6939 | 0.0040722 | 0.012182 |
| Suberate | 1.6864 | 0.00051777 | 0.00905 |
| N,N-Dimethyarginine | 1.6828 | 0.0099693 | 0.062978 |
| 5-HETE | 1.6717 | 0.0039568 | 0.076461 |
| Choline | 1.6604 | 0.015021 | 0.022034 |
| N-stearoyltaurine | 1.6468 | 0.017336 | 0.076461 |
| N-methylalanine | 1.6301 | 0.0086505 | 0.030551 |
| MG 22:6 | 1.5997 | 0.0245 | 0.076461 |
| MG 16:0 | 1.5859 | 0.010982 | 0.050798 |
| 2-aminoadipate | 1.565 | 0.012986 | 0.076461 |
| alpha-Ketoglutarate | 1.5575 | 0.0059188 | 0.062978 |
| LTB4 | 1.556 | 0.043979 | 0.111529 |
| lsoleucine | 1.457 | 0.013131 | 0.1374 |
| Uridine | 1.4199 | 0.003965 | 0.041132 |
| 14,15-DHET | 1.3945 | 0.031632 | 0.094698 |
| Azelate | 1.3885 | 0.026351 | 0.111529 |
| Dihydroorotate | 1.3556 | 0.021688 | 0.163582 |
| Alanine | 1.339 | 0.03816 | 0.094698 |
| Chenodeoxycholate-D5 | 0.89391 | 0.00048537 | 0.012182 |
| Guanosine triphosphate | 0.66217 | 0.026161 | 0.111529 |
| Oleate | 0.63233 | 0.011248 | 0.062978 |
| Glutamate | 0.60861 | 0.044648 | 0.192783 |
| Succinate | 0.54986 | 0.023552 | 0.163582 |
| PGE3 | 0.53106 | 0.021604 | 0.094698 |
| Lauroylcarnitine | 0.46379 | 0.0034768 | 0.001624 |
| Pyruvate | 0.45932 | 1.27E-05 | 0.000918 |
| Malate | 0.42024 | 0.00016139 | 0.000528 |
| Myristoylcarnitine | 0.31774 | 2.48E-07 | 0.000528 |
| Beta-hydroxybutyrate | 0.28565 | 3.28E-07 | 0.000528 |
| Palmitoylcarnitine | 0.18655 | 6.09E-08 | 0.000528 |

**Supplementary Figure 3**

**Supplementary Figure 3. Pathway enrichment analysis of age-associated metabolites** **in male kidneys (24 vs 4 months).** This figure illustrates the metabolic pathways significantly enriched among metabolites altered between 24- and 4-month-old male mice. Pathway enrichment was performed using over-representation analysis based on the list of significantly changed metabolites (p < 0.05) identified from pairwise age comparisons. Each bar represents a metabolic pathway, with the enrichment ratio shown on the x-axis, reflecting the proportion of altered metabolites relative to the total number of metabolites annotated within that pathway. Higher enrichment ratios indicate stronger over-representation. Bars are color-coded according to the corresponding p-values, with deeper red tones representing pathways with higher statistical significance, and lighter yellow/orange colors indicating less significant enrichment.

**Supplementary Figure 4**

**Male 4M**

**Male 12M**

**Male 24M**

**Supplementary Figure 4.** Heatmap illustrating the metabolites that were significantly altered during renal aging in male mice and their relative abundances across 4-, 12-, and 24-month age groups. The color gradient reflects relative abundance, with red indicating higher levels and blue indicating lower levels. n=10 (4M); n=8 (12M); n=10 (24M).

**Supplementary Table 7.** The list of significantly altered renal metabolites identified between female and male mice at 4 months of age.

| Female Vs Male -4M | FC | p_value | q-value |
| --- | --- | --- | --- |
| Lyso PE 20:1 | 1.4473 | 0.014309 | 0.959512 |
| Lyso PA 16:0 | 1.7356 | 0.026648 | 0.959512 |
| N-Acetylserine | 0.62355 | 0.03986 | 0.959512 |
| Androsterone glucuronide | 0.60041 | 0.048898 | 0.959512 |

**Supplementary Table 8.** The list of significantly altered renal metabolites identified between female and male mice at 12 months of age.

| Female Vs Male-12m | FC | p_value | q_value |
| --- | --- | --- | --- |
| Lyso PC 18:2 | 2.5819 | 0.041184 | 0.873697 |
| Caprate | 2.2668 | 0.039065 | 0.873697 |
| Hippurate | 2.1868 | 0.019449 | 0.596795 |
| Guanosine | 2.0319 | 0.019796 | 0.649744 |
| Taurocholate | 1.82 | 0.023015 | 0.649744 |
| Taurochenodeoxycholate | 1.8166 | 0.034924 | 0.596795 |
| 2-oxoadipate | 1.6463 | 0.0061838 | 0.538462 |
| Suberate | 1.5033 | 0.01633 | 0.596795 |
| Myristoylcarnitine | 0.60881 | 0.0011244 | 0.430769 |
| Decanoylcarnitine | 0.5283 | 0.010765 | 0.596795 |
| Glycerophosphocholine | 0.51367 | 0.012177 | 0.596795 |
| Lauroylcarnitine | 0.46331 | 0.0040289 | 0.596795 |

**Supplementary Table 9.** The list of significantly altered renal metabolites identified between female and male mice at 24 months of age.

| Female Vs Male-24M | FC | p_value | q_value |
| --- | --- | --- | --- |
| Indoleacetylglutamine | 2.38 | 0.0043053 | 0.204279 |
| Serine | 2.2653 | 0.004932 | 0.277254 |
| leucine | 2.159 | 0.00033921 | 0.055847 |
| Pimelylcarnitine | 1.9633 | 0.0045847 | 0.204279 |
| Phosphoserine | 1.897 | 0.012636 | 0.277254 |
| Docosahexaenoate | 1.7164 | 0.0094463 | 0.277254 |
| N-methylarginine | 1.6671 | 0.003901 | 0.175597 |
| Taurocholate | 1.592 | 0.017861 | 0.204279 |
| Indole 3-acetate | 1.5327 | 0.0030655 | 0.128245 |
| Glutamine | 1.5144 | 0.015338 | 0.277254 |
| Indole 3-lactate | 1.4828 | 0.014707 | 0.277254 |
| N-Methyglutamate | 0.7757 | 0.01652 | 0.242379 |
| Creatinine | 0.74963 | 0.035173 | 0.403679 |
| leucylglycine | 0.73484 | 0.042939 | 0.403679 |
| N-methylalanine | 0.66968 | 0.030338 | 0.429321 |
| Cysteine | 0.64533 | 0.048975 | 0.128245 |
| MG 22:6 | 0.61103 | 0.01074 | 0.175597 |
| N-Acetylphenylalanine | 0.60367 | 0.021621 | 0.403679 |
| Hypoxanthine | 0.53996 | 0.033005 | 0.277254 |
| Lyso PA 16:0 | 0.52028 | 0.047628 | 0.429321 |
| Lyso PE 20:5 | 0.5042 | 0.0070612 | 0.204279 |
| Uridine triphosphate | 0.49904 | 0.0061646 | 0.277254 |
| Lyso PE 18:3 | 0.47301 | 0.030675 | 0.384322 |
| MG 18:1 | 0.40622 | 0.02108 | 0.277254 |
| Lyso PA 18:0 | 0.38661 | 0.00017484 | 0.055847 |
| Carnitine | 0.35065 | 0.00064509 | 0.060639 |
| N-Acetylglutamate | 0.34069 | 2.69E-08 | 0.002501 |
| 3-Hydroxybutyrylcarnitine | 0.30648 | 0.0010874 | 0.069517 |

**Supplementary Figure 5**

**24 months**

**4 months**

**12 months**

**♀︎**

**♂︎**

**♀︎**

**♂︎**

**♀︎**

**♂︎**

**Supplementary Figure 5.** Heatmap illustrating the significantly regulated renal metabolites and their relative abundances across 4-, 12-, and 24-month-old female and male mice. The color gradient reflects relative abundance, with red indicating higher levels and blue indicating lower levels. n=10 (4M); n=8 (12M); n=10 (24M)

**Supplementary Figure 6**

**Supplementary Figure 6**. **Venn diagram illustrating the overlap of metabolites significantly associated with age, sex, and the age×sex interaction in mouse kidney metabolome.** Metabolites showing significant differences were identified using two-way ANOVA with factors sex and age group (4, 12, and 24 months) applied to log-transformed metabolite intensities. False discovery rate (FDR) correction was applied using the Benjamini–Hochberg method, and metabolites with q < 0.05 were considered significant. The Venn diagram indicates the number of metabolites uniquely associated with aging (Age), biological sex (Sex), or the age-by-sex interaction (Interaction), as well as shared metabolites influenced by two or more factors. This visualization highlights the relative contribution and overlap of age and sex in shaping the kidney metabolomic landscape.

**Supplementary Table 10.** The metabolites list that was significantly associated with Page, Psex, and the Page×sex interaction was identified using a two-way ANOVA with sex and age group (4, 12, and 24 months) as fixed factors.

| S.No. | Category | Metabolite |
| --- | --- | --- |
| 1 | Age_only | PGE2 |
| 2 | Age_only | LTB4 |
| 3 | Age_only | TXB2 |
| 4 | Age_only | PGE3 |
| 5 | Age_only | Myristate |
| 6 | Age_only | Palmitate |
| 7 | Age_only | Stearate |
| 8 | Age_only | Oleate |
| 9 | Age_only | X2.Hydroxyhexanoate |
| 10 | Age_only | Azelate |
| 11 | Age_only | Sebacate |
| 12 | Age_only | X8.oxoguanine |
| 13 | Age_only | Deoxyguanosine |
| 14 | Age_only | Purine |
| 15 | Age_only | Uracil |
| 16 | Age_only | Urate |
| 17 | Age_only | Uridine |
| 18 | Age_only | alpha.Ketoglutarate |
| 19 | Age_only | Beta.hydroxybutyrate |
| 20 | Age_only | Malate |
| 21 | Age_only | Methylglycine |
| 22 | Age_only | Myoinositol |
| 23 | Age_only | Succinate |
| 24 | Age_only | Lactate |
| 25 | Age_only | Lauroylcarnitine |
| 26 | Age_only | Myristoylcarnitine |
| 27 | Age_only | Palmitoylcarnitine |
| 28 | Age_only | X2.methylhippurate |
| 29 | Age_only | X3.methylhistidine |
| 30 | Age_only | N.glycylproline |
| 31 | Age_only | N.methylarginine |
| 32 | Age_only | Gamma.Aminobutyrate |
| 33 | Age_only | N.Methyglutamate |
| 34 | Age_only | N.Acetylaspartylglutamate |
| 35 | Age_only | Argininosuccinate |
| 36 | Age_only | Betaine |
| 37 | Age_only | Choline |
| 38 | Age_only | lsoleucine |
| 39 | Age_only | Glutamine |
| 40 | Age_only | Taurochenodeoxycholate |
| 41 | Age_only | Glycocholate |
| 42 | Age_only | Glycochenodeoxycholate |
| 43 | Age_only | Indole.3.acetate |
| 44 | Age_only | N.Acetylphenylalanine |
| 45 | Age_only | Phenylalanine |
| 46 | Age_only | Tyrosine |
| 47 | Age_only | Glutathione |
| 48 | Age_only | N.N.Dimethyarginine |
| 49 | Age_only | Glycerophosphocholine |
| 50 | Age_only | N.Acetylglutamine |
| 51 | Age_only | Creatinine |
| 52 | Age_only | N.Acetylserine |
| 53 | Age_only | S.MethylCysteine |
| 54 | Age_only | S.Sulfocysteine |
| 55 | Age_only | Valine |
| 56 | Age_only | Cotinine.N.oxide |
| 57 | Age_only | Pyruvate |
| 58 | Age_only | Hippurate |
| 59 | Age_only | Guanidinoacetate |
| 60 | Age_only | p.cresol.glucuronide |
| 61 | Age_only | Homocysteine |
| 62 | Age_only | Lyso.PC.18.1 |
| 63 | Age_only | Lyso.PE.18.3 |
| 64 | Age_only | Lyso.PE.20.1 |
| 65 | Age_only | Lyso.PE.20.5 |
| 66 | Age_only | MG.16.1 |
| 67 | Age_and_Sex Interaction | N.Acetylglutamate |
| 68 | Age_and_Sex Interaction | leucine |

**Supplementary Table 11.** Demographic characteristics of men and women**.**

| **Group** | **N** | **Age (Mean ± SD)** | **GFR (Mean ± SD)** | **Race Summary** |
| --- | --- | --- | --- | --- |
| **Young Female** (≤45 yrs) | 5 | 38.8 ± 4.0 | 100.4 ± 6.5 | 4 Black or African American, 1 Asian |
| **Old Female** (>45 yrs) | 6 | 68.5 ± 7.9 | 55.5 ± 23.8 | 4 Black, 1 Asian+Black, 1 White |
| **Young Male** (≤45 yrs) | 5 | 32.8 ± 7.6 | 95.3 ± 18.3 | 1 Asian, 1 Declined, 1 Black, 2 Unknown |
| **Old Male** (>45 yrs) | 5 | 72.2 ± 4.9 | 46.8 ± 8.7 | 3 Black, 1 White, 1 None of the Above |

Demographic characteristics of men and women. Mean values (SD) of age and glomerular filtration (GFR) rates are shown
